# The Regulated Secretory Pathway in CD4^+^ T cells Contributes to Human Immunodeficiency Virus Type-1 Cell-to-Cell Spread at the Virological Synapse

**DOI:** 10.1371/journal.ppat.1002226

**Published:** 2011-09-01

**Authors:** Clare Jolly, Sonja Welsch, Stefanie Michor, Quentin J. Sattentau

**Affiliations:** 1 MRC Centre for Medical Molecular Virology, Division of Infection and Immunity, University College London, London, United Kingdom; 2 Wellcome Trust Centre for Human Genetics, Division of Structural Biology, University of Oxford, Oxford, United Kingdom; 3 Structural and Computational Biology Unit, European Molecular Biology Laboratory, Heidelberg, Germany; 4 The Sir William Dunn School of Pathology, University of Oxford, Oxford, United Kingdom; Northwestern University, United States of America

## Abstract

Direct cell-cell spread of Human Immunodeficiency Virus type-1 (HIV-1) at the virological synapse (VS) is an efficient mode of dissemination between CD4^+^ T cells but the mechanisms by which HIV-1 proteins are directed towards intercellular contacts is unclear. We have used confocal microscopy and electron tomography coupled with functional virology and cell biology of primary CD4^+^ T cells from normal individuals and patients with Chediak-Higashi Syndrome and report that the HIV-1 VS displays a regulated secretion phenotype that shares features with polarized secretion at the T cell immunological synapse (IS). Cell-cell contact at the VS re-orientates the microtubule organizing center (MTOC) and organelles within the HIV-1-infected T cell towards the engaged target T cell, concomitant with polarization of viral proteins. Directed secretion of proteins at the T cell IS requires specialized organelles termed secretory lysosomes (SL) and we show that the HIV-1 envelope glycoprotein (Env) localizes with CTLA-4 and FasL in SL-related compartments and at the VS. Finally, CD4^+^ T cells that are disabled for regulated secretion are less able to support productive cell-to-cell HIV-1 spread. We propose that HIV-1 hijacks the regulated secretory pathway of CD4^+^ T cells to enhance its dissemination.

## Author Summary

Human Immunodeficiency Virus type-1 (HIV-1), the cause of the global AIDS pandemic, primarily infects CD4^+^ T lymphocytes and efficient replication necessitates transmission of infection to susceptible neighbouring cells. To ensure that the necessary components are transported to the correct location in the cell and at the right time, viral assembly is carefully controlled. To do this, HIV-1 preferentially assembles and buds at sites of cell-cell contact called virological synapses. T lymphocytes employ a specialized trafficking pathway known as the regulated secretory pathway that is activated following cell-cell contact. Here we show that HIV-1 proteins associate with cellular compartments that are involved in regulated secretion and that CD4^+^ T cells from patients with a genetic defect in this pathway are less able to support spreading infection. We conclude that HIV-1 hijacks elements of the T cell regulated secretory pathway to promote T cell-to-T cell transmission at virological synapses. This study provides the first evidence that lymphotropic viruses can hijack regulated secretion to enhance pathogenesis and may open up new opportunities for rational drug design to help combat HIV/AIDS.

## Introduction

Viral replication is a complex series of well-orchestrated events culminating in the release of progeny virions from infected cells. For efficient viral production, all components must be mobilized to the site of assembly in a coordinated manner, achieved by enlisting components of cellular transport pathways. It is becoming increasingly clear that cell-to-cell spread is an important mechanism of viral dissemination [1] and has a number of advantages for viruses, including more rapid and efficient uptake by permissive target cells. However, this process requires an exquisite level of regulation to polarize virus assembly and release towards engaged target cells at sites of cell-cell contact. Direct spread of Human Immunodeficiency Virus type-1 (HIV-1) between CD4^+^ T cells takes place across a supramolecular structure called the “virological synapse” (VS) [2]. Another mode of HIV-1 spread between immune cells is via membrane nanotubes [3], although these structures form less frequently than VS and so probably contribute less to viral dissemination [3], [4]. The VS was so named because of structural and functional similarity to the immunological synapse (IS) that evolves between an antigen presenting cell and a T cell [5]. The VS is characterised by rapid, actin-mediated recruitment of the HIV-1 entry receptors (CD4 and a chemokine receptor) and adhesion molecules on the target cell, synchronous with polarization of the HIV-1 envelope glycoprotein (Env), the Gag polyprotein and integrins in the infected cell. Within the HIV-1^+^ infected cell, Env and Gag polarization is actin- and tubulin-dependent, and requires lipid raft integrity [6], [7], [8], implying that viral proteins are actively recruited to the site of cell-cell contact. Indeed, live cell imaging visualizing *de novo* virus assembly at the VS has shown recruitment of Gag to sites of cell-cell contact [9]. By contrast, the pathway of Env trafficking in infected T cells and the molecular mechanisms underlying active Env enrichment at the VS remain poorly understood. Once at the VS, cell-to-cell spread of HIV-1 is by polarized assembly and budding of virions into the synaptic cleft and subsequent fusion with the target cell plasma membrane either at the cell surface or from within an endosomal compartment [9], [10].

Polarized secretion at the IS has been well studied in CD8^+^ T cells and is directed by antigen-dependent reorientation of the microtubule organizing center (MTOC) to sites of cell-cell contact, and the regulated movement and release of SL towards the engaged target cell [11]. By contrast, little is known about regulated secretion in CD4^+^ T cells [12], [13]. MTOC polarization at the IS occurs in CD4^+^ T cells [14], and the surface expression of membrane-associated proteins such as CTLA-4 (a negative regulator of T cell activation) and FasL (a pro-apoptotic receptor) is likely to be under the same strict temporal and spatial control orchestrated by the regulated secretory pathway in response to T cell receptor (TCR) stimulation in both lymphocyte types [15], [16], [17], [18], [19], [20]. Polarized secretion of cytokines such as IL-2 and interferon-γ is also activated at the CD4^+^ T cell IS, thereby focusing the exocytosis of specific soluble proteins towards target cells following cell-cell contact [21], [22].

Directed egress of HIV-1 at the VS is reminiscent of polarized secretion by T cells at the IS that is coordinated by the regulated secretory pathway (reviewed in [11], [12], [13]). We therefore hypothesized that HIV-1 may harness elements of this pathway to orchestrate egress from CD4^+^ T cells and promote cell-to-cell spread. Here we confirm that CD4^+^ T cells contain a compartment that is similar to SL in CD8^+^ T cells and show that the T cell regulated secretory pathway contributes to HIV-1 cell-to-cell dissemination, providing the first evidence that lymphotropic viruses can hijack regulated secretion to enhance pathogenesis.

## Results

### The HIV-1 VS has a regulated secretion phenotype

**Figure 1:**
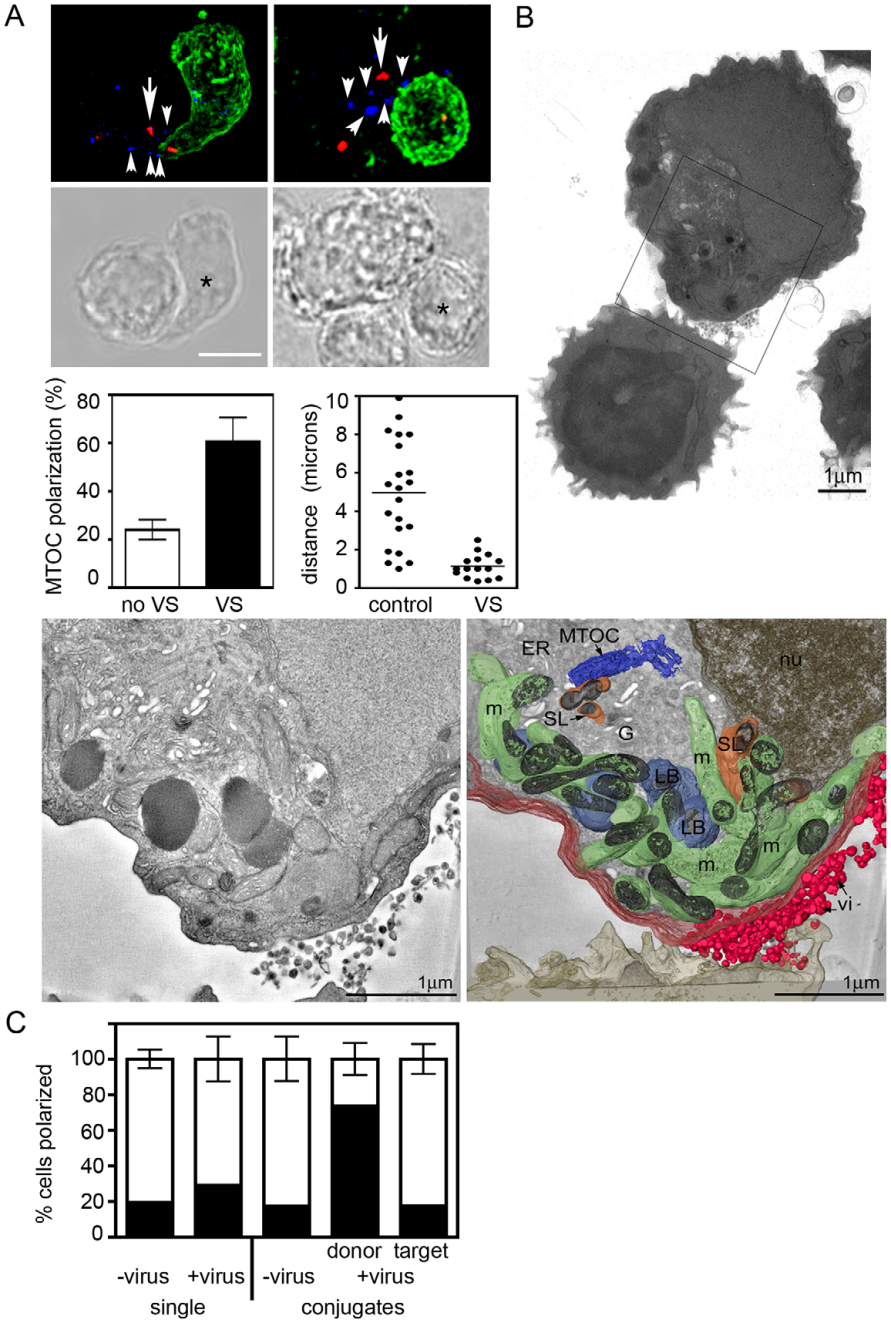
The HIV VS has a regulated secretion phenotype. **A**) *The MTOC aligns proximal to the VS in the HIV-1 infected T cell*. Primary CD4^+^ T cells infected with the HIV-1 strain BaL were mixed with uninfected autologous target CD4^+^ T cells that were pre-stained with the non-inhibitory anti-CD4 mAb L120 (green) and cells were incubated on poly-L-lysine-coated coverslips for 1 h at 37°C. Conjugates were fixed, permeabilized and stained for intracellular HIV-1 Env (blue) and γ-tubulin to label the centrosome (red). Two representative 3D reconstructed projections (top panels) and the corresponding DIC images (lower panels) are shown of conjugates formed between an HIV-1 infected T cell and an uninfected target cell (asterisk) showing polarization of the MTOC in the infected cell (indicated by the large arrow). Clusters of HIV-1 Env in the infected cell (indicated by small arrowheads) are located near to the cell-cell interface and around the MTOC. Scale bar = 5 µm. MTOC polarization was quantified (lower left panel) in conjugates that had formed a VS (black bar, defined by enrichment of viral proteins in infected cells at the contact site) or had not formed a VS (white bar, no HIV-1 enrichment) and the MTOC was scored as polarized if it was aligned at the interface-proximal third in the HIV-1 infected cell. Data are from three independent experiments and the error bars represent the SEM. The right hand graph shows the average distance of the MTOC from the plasma membrane at the VS or at the contact site between two uninfected cells (control). Data are from three independent experiments. **B**) *Alignment of secretory organelles and MTOC proximal to the VS in the HIV-1 infected T cell.* Infected Jurkat T cells were mixed with primary CD4^+^ T cells, fixed and embedded. Top panel is a VS overview image. The box indicates the region of the thick section shown in the 3D surface rendering (lower panel) of a tomogram, reconstructed from two sequential tilt series taken from 300 nm sections. Redistribution of secretory organelles (endoplasmic reticulum, ER, not pseudocoloured), secretory lysosomes (SL, orange), Golgi (G, not pseudocoloured), mitochondria (m, green) and lipid bodies (LB, pale blue) and MTOC (blue), towards the site of virus particle (vi, red) release in the HIV-1 infected T cell. **C**) *Quantification of cell polarization.* Conjugates between HIV-1 infected cells and target T cells with obvious virus budding (n = 31), or conjugates between two cells without virus (n = 55) were examined by EM and quantified for whether or not cellular organelles were polarized to the site of cell-cell contact. Cells were defined as polarized only when most of the cytoplasm and all the mitochondria, lipid bodies and lysosomes were localized to the contact site. Polarized  =  black, unpolarized  =  white. Target cell  =  uninfected cell, donor  =  HIV-1 infected cell. Single unconjugated cells showing budding virus (n = 96) were also scored for whether organelles were polarized to the site of virus budding, or in the case of single cells without budding virus (n = 216) whether any cellular polarization was evident. Data are from two independent experiments in which two separate grids were examined. See also Fig. 1 and Video S2–S4.

A characteristic feature of IS formation and regulated secretion by T cells at sites of cell-cell contact is polarization of the microtubule organizing center to the conjugate interface, aligning the secretory apparatus of the T cell proximal to the contact site and directing secretion towards the engaged target cell [14], [23], [24], [25], [26], [27], [28]. To investigate whether similar polarization occurs at the VS we performed immunofluorescence staining and confocal microscopy and quantified MTOC localization in conjugates between HIV-1-infected primary CD4^+^ T cells and autologous target T cells. MTOC polarization was examined in conjugates that had evolved VS (identified by the enrichment of HIV-1 proteins in the infected T cell at the contact site) and was compared to conjugates between infected and uninfected cells that had not formed VS (i.e. did not show HIV-1 enrichment at the contact site). In a manner analogous to the T cell IS, we observed statistically significant polarization of the MTOC within HIV-1-infected primary CD4^+^ T cells towards engaged autologous target T cells at the VS (Fig. 1a). Alignment of the MTOC in infected cells proximal to the site of cell-cell contact occurred in 60% ±10% of VS (n = 31, *p* = 0.004) compared to 24% ±4% of conjugates that were in contact but had not formed a VS (n = 40). Intracellular clusters of HIV-1 Env were observed near to the polarized MTOC and close to the cell-cell interface (Fig. 1a), although the necessity for methanol permeabilization for γ-tubulin labeling in T cells resulted in the loss of most surface Env signal. On average the MTOC within the HIV-1 infected cell was located 1 µm from the plasma membrane at the VS (Fig. 1a) whereas HIV-1 independent contacts between two uninfected T cells did not induce polarization and the MTOC was on average 5 µm from the cell-cell interface. Thus MTOC polarization at sites of cell-cell contact correlates with VS assembly in HIV-1 infected T cells.

The tubulin cytoskeleton is implicated in promoting cell-to-cell dissemination of HIV-1 at the VS [7]. We therefore hypothesized that by analogy with the IS, MTOC polarization might align viral antigen-containing secretory organelles at the VS and organize the microtubule network for efficient HIV-1 egress via directional budding. In order to visualize cellular secretory components at the VS in more detail we performed ultrastructural analysis by electron microscopy (EM) and tomography. Fig. 1b shows an example of a conjugate formed between an HIV-1-infected T cell and an uninfected CD4^+^ T cell in which virus particles are concentrated at the VS. Reconstruction of a 3D model of the contact zone between the cells revealed clustering in the infected cell of organelles diagnostic for polarization of the T cell secretory apparatus: multiple mitochondria, lysosomes, lipid bodies and the MTOC [23], [24], [25], [29](Fig. 1b and Video S1). To investigate whether polarization was specifically associated with the VS, we identified conjugates between HIV-1 infected T cells and uninfected T cells with HIV-1 budding at the interface and quantified whether the cells showed a polarized phenotype in relation to the contact site (Fig. 1c, Fig. S1 and Video S2–S4). A cell was defined as polarized when most of the cytoplasm was on one side of the nucleus and all the mitochondria, lysosomes and lipid bodies were associated with the cytoplasm-enriched area (Fig. S1). Using this relatively stringent definition of polarization we observed complete polarization of organelles towards the cell-cell interface in 75% of HIV-1 infected T cells with budding virus at the VS. Polarization was specific to the HIV-1 infected cell since it was rarely seen in the conjugated target cell (18%) or in conjugates between two uninfected cells (17%)(Fig. 1c). Notably, we did not identify conjugates in which organelles were polarized away from the contact interface. Single (unconjugated) HIV-1 infected T cells with budding virus were also evident but in these cells polarization of organelles at the budding site was seen only 30% of the time, indicating that in the majority of infected cells adopt a polarized phenotype in response to cell-cell contact.

### Env colocalizes with SL-associated proteins in CD4^+^ T cells and at the VS

**Figure 2:**
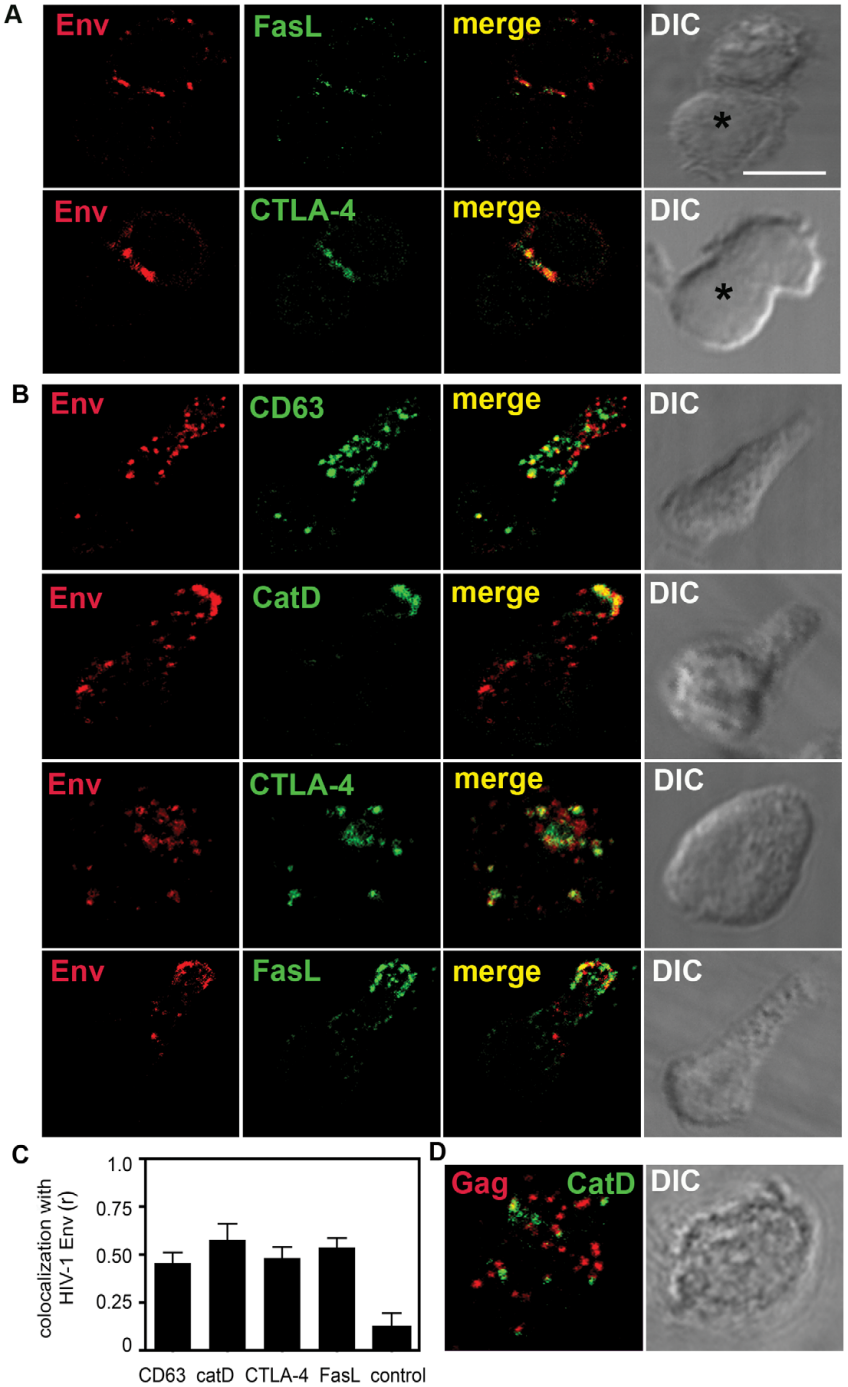
HIV-1 Env associates with SL-related organelles in CD4^+^ T cells. **A**) *SL-associated proteins are co-enriched with Env at the VS*. HIV-1 infected CD4^+^ T cells were mixed with uninfected autologous target T cells and incubated on coverslips at 37°C for up to 2 h. Conjugates were fixed, permeabilized and stained for Env (red) and FasL or CTLA-4 (green). Images are single *xy* sections taken through the middle of a conjugate with the corresponding DIC image. The target cell is indicated with an asterisk. Areas of colocalization are yellow. Scale bar = 5 µm. **B**) *HIV-1 Env colocalizes intracellularly with SL-associated proteins.* Single CD4^+^ T cells infected with HIV-1 were fixed, permeabilized and stained for intracellular Env (red) and CD63, cathepsin D, CTLA-4 or FasL (green). Primary antibodies were detected with species or isotype-specific conjugated secondary antibodies. Images are 3D reconstructions of serial *z-*sections taken by LSCM with the corresponding merged and DIC images shown. **C**) Quantification of colocalization between Env and CD63 (n = 21), cathepsin D (n = 25), CTLA-4 (n = 20) and FasL (n = 20) are shown. As a control, colocalization between Env and the mitochondrial protein ATP synthase subunit β was also quantified. For each infected cell, quantification of colocalization was performed on a single *xy* slice extracted from the corresponding *z* series and the Pearson’s correlation coefficient (r) was calculated. The average r values are graphed and error bars show the SEM from four experiments performed using six individual donors. **D**) *HIV-1 Gag does not colocalize with cathepsin D.* HIV-1 infected CD4^+^ T cells were fixed, permeabilized and stained for HIV-1 Gag (red) and cathepsin D (green). The corresponding DIC image is shown.

To investigate whether the regulated secretory pathway maybe implicated in HIV-1 egress at the T cell VS, we incubated HIV-1 infected primary CD4^+^ T cells with uninfected autologous CD4^+^ T cells and examined conjugates for VS formation by immunofluorescence labelling (IF) and laser scanning confocal microscopy (LSCM)[2]. Similar to our previous observations using infected immortalized T cell lines [2], we found that polarization of Env to the site of cell-cell contact occurred in approximately 30% of conjugates examined (Fig. 2a). CTLA-4 and FasL are trafficked in T cells by regulated secretion in secretory lysosomes (SL), and coordinate with Env polarization, CTLA-4 and FasL were also enriched at the VS (Fig. 2a) with 63% ±9% of VS showing co-localization of CTLA-4 with Env at the contact site. FasL staining at the VS was generally weak, consistent with observations by others, probably reflecting both the relatively short half-life of FasL in SL [30] and the recognition by mAb NOK-1 of only newly synthesized intracellular protein, which represents a subset of total FasL [15].

The concomitant enrichment of CTLA-4 and FasL at the VS, together with the previous observation that the endosome-lysosome marker CD63 is also recruited to the VS [31] suggests that HIV-1 proteins may be directed to cell-cell contact zones in association with elements of the CD4^+^ T cell regulated secretory pathway. To address whether Env may be associating with SL-like compartments in HIV-1 infected CD4^+^ T cells, we first confirmed that CD4^+^ T cells contain a secretory compartment that is antigenically similar to SL described in CD8^+^ T cells and NK cells [15], [17], [32] by performing colocalization analysis with CD63, CTLA-4 and FasL (Fig. S2a, b and S3). Next, we examined the localization of HIV-1 proteins with these markers in primary CD4^+^ T cells from healthy donors. Cells were analyzed for markers of SL and Env by LSCM at the peak of virus infection (day 7–10 post-infection). Intracellular Env colocalized with SL-associated proteins cathepsin D (r = 0.58), CTLA-4 (r = 0.48), FasL (r = 0.54) and CD63 (r = 0.46) but not with a control antibody specific for the mitochondrial protein ATP synthase subunit β (r = 0.12) (Fig. 2b and c). By contrast we observed very little colocalization of HIV-1 Gag with cathepsin D (Fig. 2d) in agreement with Env and Gag trafficking independently to the plasma membrane in infected T cells. Taken together these data suggest that HIV-1 Env may localize to an intracellular compartment with SL properties, referred to herein as an SL-related organelle (SLRO) in the context of HIV-1 infection and that Env enrichment at the VS may be associated with elements of the regulated secretion pathway in CD4^+^ T cells.

### Defects in regulated secretion: Chediak Higashi Syndrome

**Figure 3:**
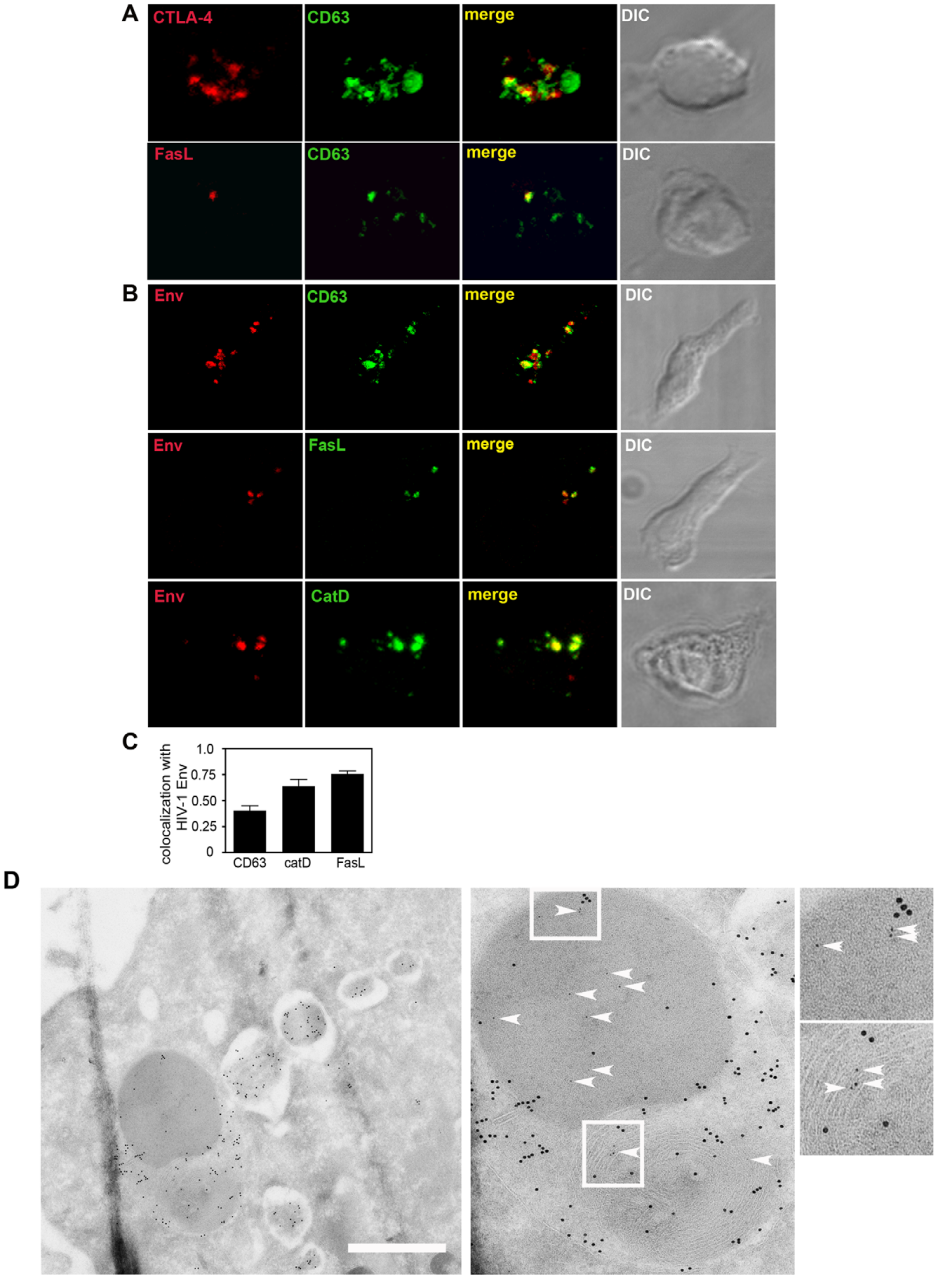
HIV-1 Env colocalizes with enlarged SL-related organelles in CHS cells. **A**) *CD4^+^ T cells from CHS individuals contain enlarged lysosomes*. CD4^+^ T cells were activated for 24 h with anti-CD3 mAb at 7–14 days post-stimulation, fixed, permeabilized and stained by immunofluorescence for CD63, CTLA-4 and FasL. Images are 3D reconstructed *z*-series, areas of colocalization appear yellow and the corresponding DIC images are shown. Enlarged CD63^+^ SLs are present without CD3 activation, but CTLA-4 and FasL expression are increased following activation (data not shown). **B**) *HIV-1 Env colocalizes with SL in CHS CD4^+^ T cells.* CD4^+^ T cell clones were infected with HIV-1 and 10–12 days post-infection the cells were fixed, permeabilized and stained by IF for Env (red) and CD63, FasL or cathepsin D (green). Images are 3D reconstructed *z*-series and areas of colocalization are yellow. **C**) Quantification of colocalization (the average r value) is shown for Env and CD63 (n = 21), cathepsin D (n = 25) and FasL (n = 21) and was performed as described in Fig. 2. Data are from four independent experiments performed with three cell lines from two unrelated CHS patients. **D**) *Cryo-immuno EM of Env and cathepsin D staining in HIV-1-infected CHS cells.* CHS cells were infected with HIV-1, fixed, frozen and cryosectioned and stained with anti-cathepsin D (detected with 10 nm gold) and the HIV-1 Env mAb2G12 (detected with 5 nm gold). Left panel shows the presence of multiple, enlarged cathepsin D-positive compartments in the CHS cell. This is shown in more detail in the middle panel with Env (5 nm gold) staining highlighted with arrowheads. Two boxed regions are magnified in the right panel to more clearly show cathespin D (10 nm gold) and Env (5 nm gold and arrowheads) colocalization. Scale bar = 600 nm.

To evaluate whether the regulated secretory pathway plays any functional role in HIV-1 dissemination we examined HIV-1 infection in the context of T cells that do not have fully functional regulated secretory phenotype. Hematopoietic cells from individuals with Chediak-Higashi Syndrome (CHS), a rare, autosomal recessive condition characterized by partial albinism, prolonged bleeding time and immune dysfunction, display abnormally large CD63^+^ SL that are unable to exocytose at the plasma membrane [33]. The affected protein has been identified as the lysosomal biogenesis regulator Lyst (or CHS-1), a 400kD protein that is believed to regulate vesicle fusion/fission [33]. When examined by LSCM, IL-2-dependent CD4^+^ T cell lines derived from two unrelated individuals with CHS contained CD63^+^ compartments that were characteristically reduced in number, often enlarged and co-labelled for cathepsin D, CTLA-4 and FasL (Fig. 3a) as reported previously [32], [34]. In agreement with others [32] we also found that surface expression of CTLA-4 and CD63 was not upregulated in CHS cells even after 1 h treatment with PMA-ionomycin (MFI CTLA-4 untreated  = 2.9, 1 h = 3.0; CD63 untreated  = 212, 1 h = 210) whereas cells from normal donors rapidly increased surface expression following stimulation (Fig. S4). After 4 h stimulation a slight increase in CD63 was detected (MFI = 335)(data not shown) probably reflecting trafficking via a constitutive pathway that directs CD63 via the plasma membrane *en route* to lysosomes [35] superimposed onto the defective regulated secretory pathway. Collectively these data confirm the presence of the expected defect in CHS cells and support our data (Fig. S2) and those of others [17] that CD4^+^ T cells, like CD8^+^ T cells contain a functional SL-related compartment for regulated secretion.

### Env localizes to SL-related compartments in CHS T cells

To examine the localization of Env in cells with abnormal SL-related compartments, three CD4^+^ T cell lines from two unrelated CHS patients were infected with HIV-1 and examined by immunofluorescence LSCM. We observed a partial accumulation of Env (Fig. 3b and c), but not Gag (Fig. S5) with SL-associated proteins in CHS cells that was reproducible using different CHS-derived clones or PHA blasts, and using CXCR4 or CCR5-tropic strains of HIV-1. The presence of Env colocalizing with cathepsin D in CHS lysosomal organelles was also confirmed by ultrastructural analysis of cryosectioned HIV-1-infected CHS CD4^+^ T cells (Fig. 3d).

**Figure 4:**
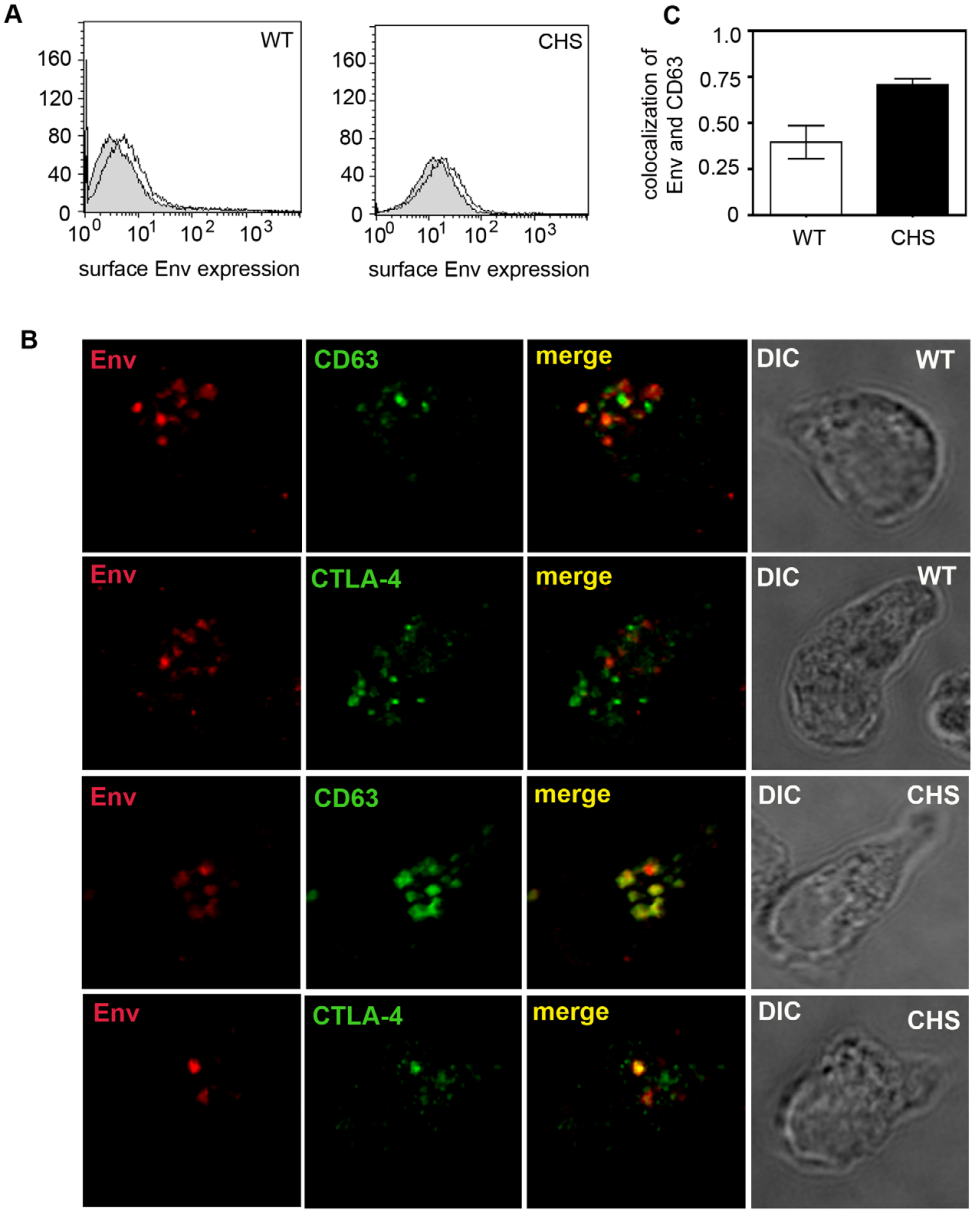
HIV-1 Env colocalizes with SL-associated proteins following endocytosis from the plasma membrane. **A**) *HIV-1 infected WT and CHS CD4^+^ T cells express HIV-1 Env at the plasma membrane, evidence of functional constitutive trafficking.* WT and CHS cells infected with HIV-1 were stained with the mAb 2G12 and anti-human conjugated-PE on ice at 7 days post-infection to label surface expressed gp120 and analyzed by flow cytometry. HIV-1 gp120 surface staining on infected cells (black line) is overlaid onto uninfected cells also stained with 2G12 (grey filled). Histograms are representative of independent experiments performed with two WT and two CHS cell lines. **B**) *Confocal analysis of endocytosed Env colocalizing with SL-associated proteins.* WT CD4^+^ T cells (top two panels) and CHS cells (lower two panels) were infected with HIV-1, incubated for 4–5 days, washed and incubated for 24 hours in media containing 10 µg/ml of the HIV-1 gp120 specific mAb IgGb12. The cells were then fixed, permeabilized and stained with anti-CD63 or anti-CTLA-4 (green). Endocytosed, intracellular Env was detected with anti-human secondary antibody (red) and CD63 and CTLA-4 with anti-mouse secondary antibody. Images are 3D reconstructed z-series representative of experiments performed with cells from two CHS patients and two WT donors. Areas of colocalization are yellow and the corresponding DIC images are shown. **C**) Quantification of colocalization between endocytosed Env and SL-associated proteins (average r value) is shown for Env and CD63 in WT cells (n = 9) and CHS cells (n = 18) and was performed as described in Fig. 2. Error bars show the SEM.

In HIV-1 infected cells Env is trafficked to the plasma membrane and subsequently endocytosed by clathrin adaptor complexes, similar to CTLA-4 [36], [37], [38], [39], [40], [41], [42]. To determine if HIV-1 Env may be associating with SL-related compartments in CD4^+^ T cells following endocytosis from the plasma membrane, we first performed surface staining and flow cytometry analysis and demonstrated that HIV-1 Env can be detected at the plasma membrane of WT and CHS cells, providing evidence either for a functional constitutive pathway from the TGN to the plasma membrane in these cells or for previous VS formation between these cells and target cells (Fig. 4a). Next, HIV-1 infected WT or CHS cells (two different donors for each cell type) were incubated with a mAb specific for the HIV-1 Env subunit gp120 for 24 h at 37°C to allow endocytosis of antibody in complex with plasma membrane exposed gp120. We used the mAb IgG1b12 that recognizes the CD4 binding site on gp120 in order to avoid detecting gp120 bound to CD4 on the surface of infected cells since endocytosed gp120-CD4 complex could be potentially sorted into a different (CD4-mediated) pathway to unliganded gp120. Taking this approach we observed strong colocalization between endocytosed HIV-1 gp120 and CD63 (r = 0.7) and between gp120 and CTLA-4 in CHS cells (Fig. 4b and c). By contrast less colocalization was evident between gp120 and SL-associated proteins in WT cells (CD63 and gp120, r = 0.39). This suggests that a proportion of HIV-1 Env can access SL-related compartments in CHS T cells after endocytosis from the plasma membrane and that Env may preferentially accumulate therein in CHS cells.

### CHS cells are less able to support a spreading HIV-1 infection

**Figure 5:**
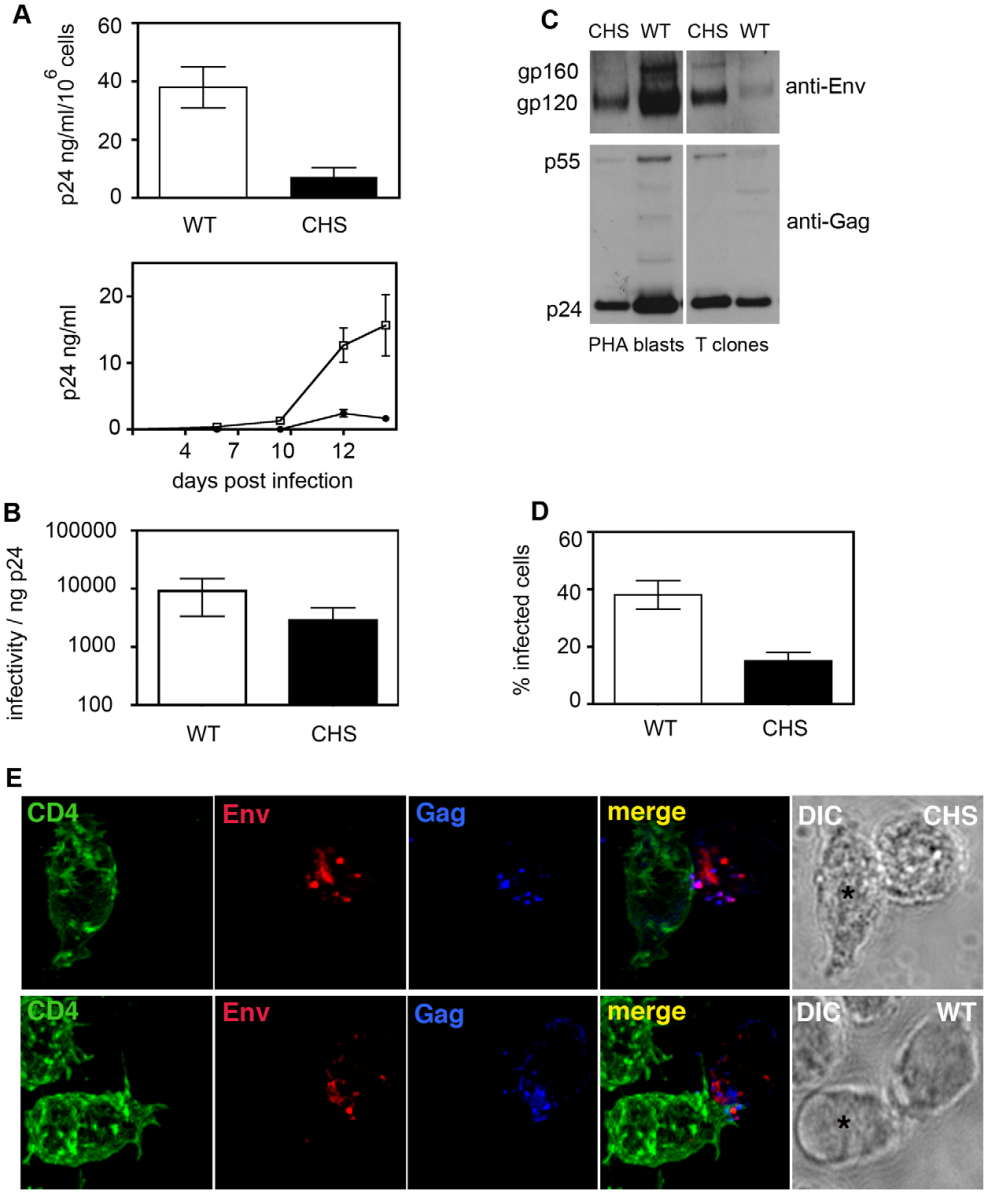
CHS CD4^+^ T cells are less able to support HIV-1 replication in culture. **A**) CHS CD4^+^ T cells (black bars) and cells from normal WT donors (white bars) were infected with HIV-1 and supernatants were taken at various days post-infection and virus release was quantified by HIV-1 Gag p24 ELISA. Values in the upper panel were normalized to viable cell count and are ng p24/ml/10^6^ cells and show Gag p24 from supernatants taken at 10–12 days post-infection. The lower panel shows the Gag p24 levels from the same experiments at all time points. Data are from four independent experiments performed using four different WT donors and three different CHS cell lines from two unrelated patients. Error bars show the SEM. **B**) *CHS cells produce infectious virus.* Cell-free supernatants were harvested at 10–12 days post-infection and viral infectivity was quantified on HeLa Tzm-bl reporter cells by luciferase assay. The TCID50 was calculated and data are shown as the TCID50 per ng of p24 to normalize for differences in the amount of virus in the supernatant. Data are from three independent experiments performed using four different WT donors and three different CHS cell lines from two unrelated patients. Error bars show the SEM. **C**) *Western blotting analysis of HIV-1 Env incorporation into purified virions*. Viral particles from HIV-1 infected cultures were purified by ultracentrifugation and concentrated 10-fold. An equal volume was loaded onto polyacrylamide gels and viral proteins were separated by SDS-PAGE. Western blotting was performed with rabbit anti-Env and rabbit anti-Gag serum and proteins were visualized by ECL. Western blots of two WT donors and two CHS patients are shown and are representative of results obtained using four WT virus samples and four CHS virus samples. One example of PHA blasts (left panels) and one example of cloned CD4^+^ T cells (right panels) are shown. Note that HIV-1 Env gp120 and unprocessed gp160 can be detected in all virus samples. **D**) *Quantification of HIV-1 infected cells.* CHS cells and WT cells were fixed, stained for HIV-1 Gag and the number of Gag^+^ infected cells was quantified by LSCM. Data are from three independent experiments with the SEM. **E**) *Confocal microscopy of a VS formed between an infected CHS T cell and a target T cell.* HIV-1 infected CHS CD4^+^ T cells were mixed with an equal number of uninfected CHS CD4^+^ T cells (prestained with the CD4 mAb L120 (green)) and conjugates were incubated for 1 h at 37°C (upper panel). Cells were then fixed, permeabilized and stained for HIV-1 Env with 2G12 (red) and HIV-1 Gag (blue) and examined by LSCM. The target cell is indicated with an asterisk on the corresponding DIC image. A representative VS formed between two WT cells is shown for comparison (lower panel).

To investigate whether defects in regulated secretion from CD4^+^ T cells correlated with a functional defect in HIV-1 egress and/or cell-to-cell spread we compared three different CHS CD4^+^ T cell lines from two unrelated donors with WT cells from at least four independent donors for their ability to support HIV-1 replication. CHS cells were appropriately matched with WT cells (clones vs PHA blasts), stimulated and activated in parallel to allow direct comparisons and infected with replication competent HIV-1. Cell-free supernatants were taken on different days post-infection and virus release was quantified by HIV-1 Gag p24 ELISA. Data from multiple infection experiments performed with different CHS and WT cells were combined and quantification of HIV-1 Gag p24 release revealed significantly less viral protein present in the culture supernatant of CHS infected cells compared to WT cells (Fig. 5a, *p* = 0.008). This was independent of cell number, growth kinetics, CD4 or HIV-1 co-receptor usage and was evident with both CCR5 and CXCR4-tropic viruses (data not shown). Quantification of viral infectivity revealed that the virus produced by CHS cells was modestly but not significantly reduced in infectivity when compared to virus released from wild-type cells (Fig. 5b). Western blotting of cell-free virus purified by ultracentrifugation from CHS and WT cultures (PHA blasts and CD4^+^ T cell clones) confirmed the presence of proteolytically processed HIV-1 gp120 in viral particles demonstrating that functional Env can be incorporated into cell-free virions (Fig. 5c); however, variation in Gag p24 and Env gp120 levels between virus samples from different donors precluded accurate analysis of the relative amount of HIV-1 Env incorporation. We did not observe any obvious increase in unprocessed Gag p55 indicating CHS cells produce proteolytically-mature cell-free virus. Although both WT and CHS cells produced infectious virus, more HIV-1-infected CD4^+^ T cells were present in WT cultures (38±5%) compared to CHS cultures (15% ±3%, *p* = 0.004)(Fig. 5d) and a trend towards less frequent cell-cell interactions and VS formation (defined as colopolarization of Env and Gag at sites of cell-cell contact) was evident with HIV-1-infected CHS cells. By contrast, conjugates between HIV-1 infected WT cells and target CD4^+^ T cells were more readily formed and polarization of viral proteins to the VS was apparent in 42% of conjugates examined (n = 57). When examined by LSCM, we noted that when VS did form between HIV-1 infected CHS cells and uninfected autologous T cells viral proteins seemed to be less polarized at sites of cell-cell contact than in WT cells (Fig. 5e); however limited patient samples meant that we were unable to quantify this observation.

**Figure 6:**
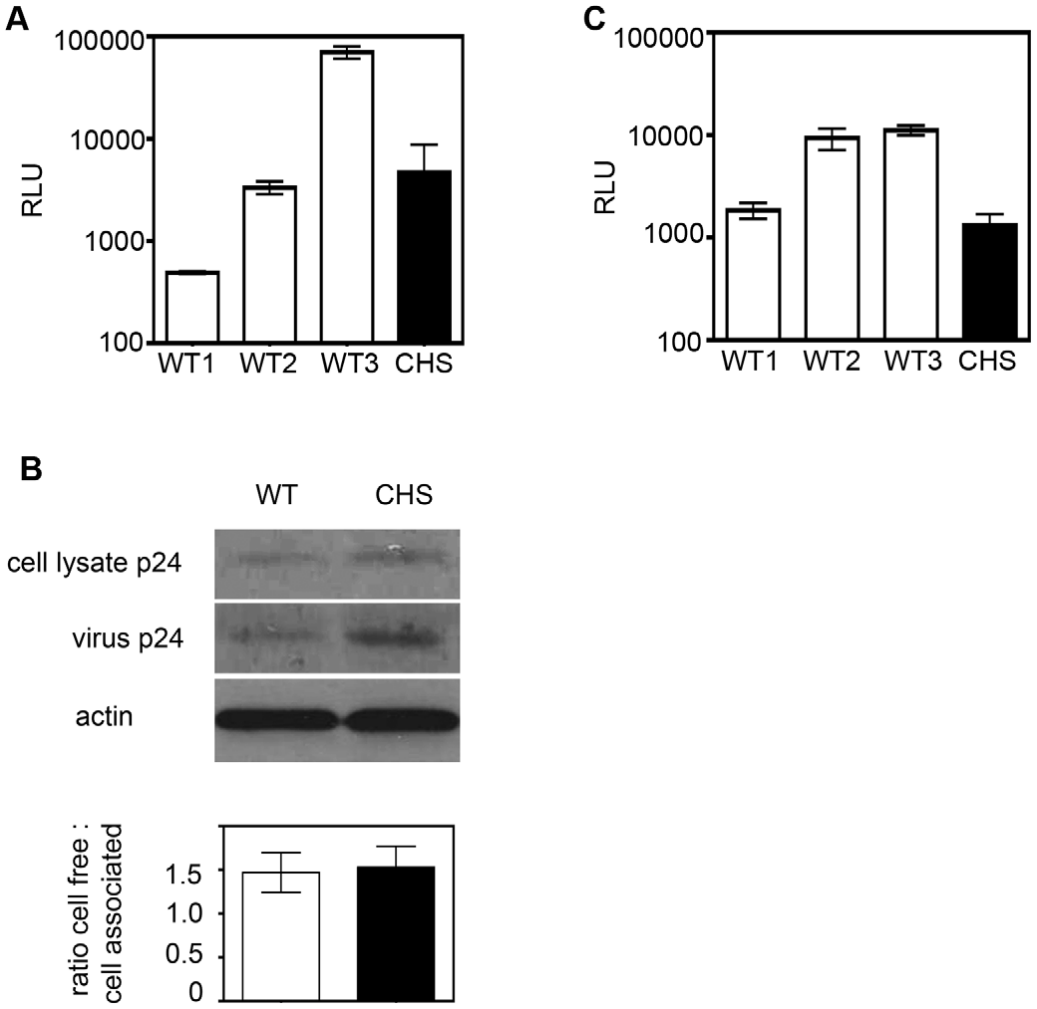
CHS cells support HIV-1 infection and Gag budding over a single- round of replication but transmit virus less efficiently by cell-to-cell spread. **A**) *CHS CD4^+^ T cells are susceptible to infection with pseudotyped virus.* 10^6^ CD4^+^ T cell blasts from three WT donors and one CHS patient were infected in duplicate with replication-defective VSV-G pseudotyped HIV-1 encoding the luciferase gene. Infection was quantified after 24 h by luminescence assay and the relative light units are shown with the SEM. Data are from two independent experiments performed with three unrelated WT donors and one CHS donor. **B**) *CHS and WT cells release equivalent amounts of Gag p24 over a single infection cycle.* 10^6^ WT and CHS CD4^+^ T cells were infected in triplicate with replication-defective VSV-G pseudotyped HIV-1 and cell and viral lysates were separated by SDS-PAGE and probed with rabbit anti-Gag by Western blotting. The relative amount of cell-free-to-cell-associated Gag was quantified using Image J. Equal loading of cell lysates was verified by probing membranes with anti-actin antibody. A representative blot is shown with quantification from three independent experiments performed with four WT donors and one CHS patient. Error bars show the SEM. **C**) *CHS cells transmit virus less efficiently to target T cells by cell-to-cell spread.* CD4^+^ T cell blasts from three WT donors and one CHS patient were infected with HIV-1 strain NL4.3 for 48 h and an equal number of donor cells were mixed with 1G5 target T cells for 24 h.1G5 cells express an HIV-1 Tat-inducible firefly luciferase reporter gene and cell-to-cell spread of HIV-1 from donor T cells to target T cells was measured by luminescence assay. Data from one of two independent experiments is shown and error bars show the SD.

The difference we detected by p24 ELISA in the amount of extracellular HIV-1 capsid protein in CHS cultures compared to WT and the difference in the number of infected cells could be caused by a block in initial infection of CHS cells, by a reduction in cell-free virus production from infected cells, or by a defect in the ability of CHS cells to efficiently disseminate infection to neighboring cells by cell-to-cell spread. To address these possibilities we first quantified infection of CHS and WT cells using single-cycle, replication defective pseudotyped HIV-1 that contains a luciferase reporter gene, allowing infection to be measured by luminescence assay without a spreading infection taking place. CHS and WT CD4^+^ T cells were incubated with virus for 4 h, unbound virus was removed by washing and infection was measured by luciferase activity after 24 h. Fig. 6a shows that both WT and CHS cells were similarly susceptible to infection with pseudotyped virus, demonstrating that CHS cells can support infection leading to proviral integration and suggesting that a block to infection is not responsible for the defect in HIV-1 replication in CHS cells.

We next addressed whether Lyst-defective cells may have a late-stage defect in cell-free virus assembly and release. CHS and WT cells were again infected with single-cycle, replication-defective pseudovirus and Gag p24 release was examined by SDS-PAGE and Western blotting. This infection assay faithfully mimics cell-free virus production over a single round of infection because although Gag virus-like particles are released from cells they are Env^-^ and so cannot initiate a spreading infection. Fig. 6b shows that Gag p24 could be detected in cell-free supernatants and in cell lysates from WT and CHS cells with no difference in the ratio of cell-free to cell-associated Gag (Fig. 6b), demonstrating that the reduced levels of infection seen with replication-competent HIV-1 is not due to a defect in Gag synthesis or a block in cell-free virus budding from CHS cells.

Finally, to directly quantify cell-to-cell spread CHS and WT CD4^+^ T cells were infected with the replication competent HIV-1 strain NL4-3, incubated for 48 h to allow viral protein expression and nascent HIV-1 production, and an equal number of infected cells were mixed with uninfected 1G5 target T cells. 1G5 cells contain an HIV-1 Tat-inducible firefly luciferase reporter gene [43] allowing infection to be quantified by luminescence assay after 24 h, a time frame in which only a single round of infection will take place [7], [8], [44], [45], [46], [47]. Coculture of 1G5 target cells with HIV-1 infected CHS donor cells gave a 10-fold reduction in RLU compared to target T cells that were incubated with WT donor cells, indicating that HIV-1 infected CHS T cells were less efficient at transmitting infectious virus by cell-to-cell spread (Fig. 6c). It should be noted that target T cells incubated with HIV-1 infected WT cells from donor 1 displayed less efficient transmission than other WT cells tested. We observed that prior to infection with HIV-1 the cells from this donor stimulated and proliferated less robustly than the others (data not shown) and this that may have impacted on the ability of these cells to transmit virus. It is also possible that these cells contained another polymorphism that may affect the efficiency of HIV-1 replication and spread.

### Defects in regulated secretion inhibit cell-to-cell spread of HIV-1

**Figure 7:**
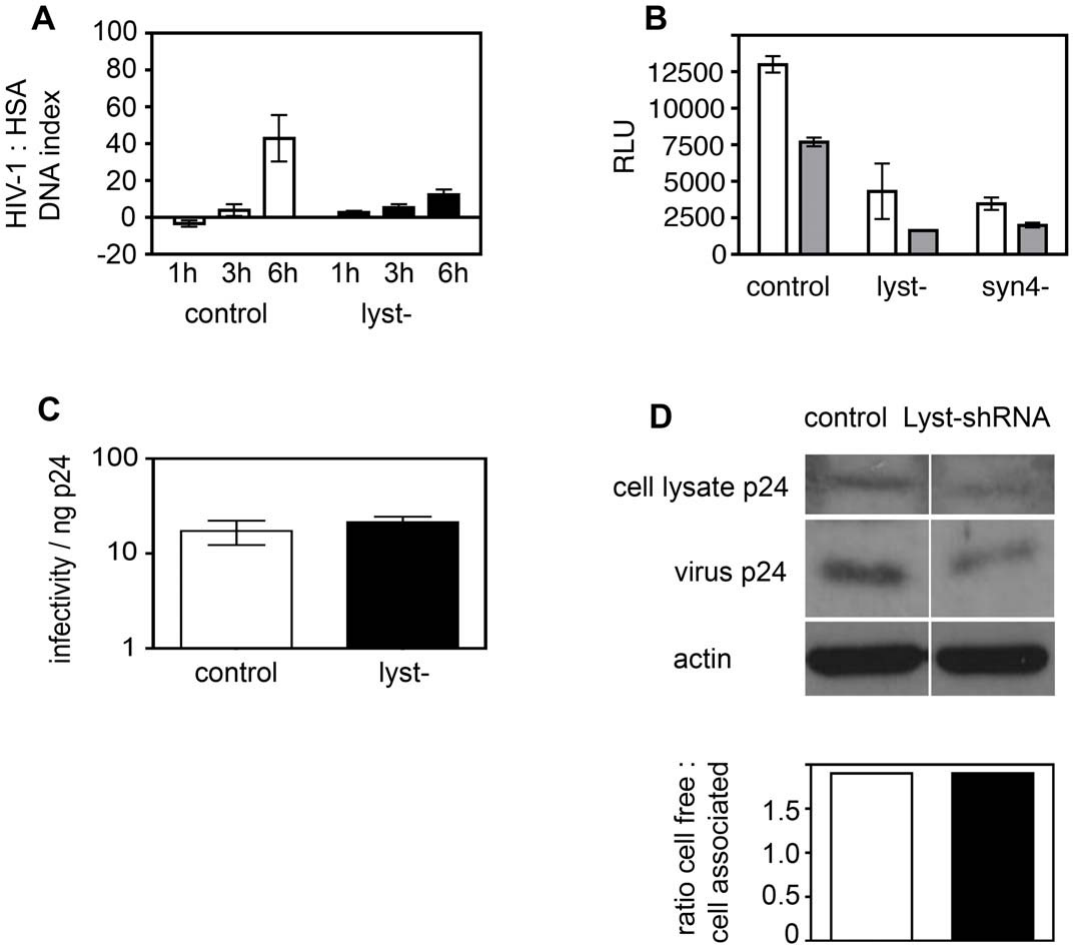
Defects in regulated secretion inhibit cell-to-cell spread of HIV-1. **A**) *Lyst modulates cell-to-cell spread of HIV-1 measured by quantitative real-time PCR*. HIV-1 infected Jurkat T cells stably expressing Lyst-shRNA (black bars) or control-shRNA (white bars) were mixed with an equal number of uninfected target T cells and incubated for 0, 1, 3 and 6 hours prior to lysis and extraction of DNA. Quantitative real-time PCR using *pol* primers was performed to quantify *de novo* HIV-1 DNA synthesis as a measure of HIV-1 cell-to-cell spread. The HIV-1 DNA copy number was normalized to human serum albumin (HSA) and is shown as the ratio of HIV-1 DNA to HSA DNA. The data obtained at t = 0 h (baseline) were subtracted from the test values. Data are from three independent experiments and error bars show the SEM. **B**) *Quantification of cell-cell spread using a luciferase reporter T cell line.* HIV-1 infected Jurkat T cells expressing control-shRNA, Lyst-shRNA or Syntaxin 4-shRNA were mixed with an equal number of uninfected 1G5 target T cells that were either untreated (white bars) pre-treated with 50 µM Zidovudine (grey bars) and incubated for 24 h. 1G5 cells express an HIV-1 Tat-inducible firefly luciferase reporter gene and virus infection was measured by luminescence. Data show the relative light units from a representative of two independent experiments and error bars show the SD. **C**) *Lyst knockdown cells produce infectious cell-free virus.* Cell-free supernatants were harvested from cells infected with replication competent HIV-1 at 7 days post-infection and viral infectivity was quantified on HeLa Tzm-bl reporter cells by luciferase assay. The TCID50 was calculated and data are shown as the TCID50 per ng of p24 to normalize for any differences in the viral content of supernatants. Data are from three independent experiments and show the SEM. **D**) *Lyst and control knockdown cells produce equivalent amounts of p24 over a single infection cycle.* Lyst-shRNA cells and control-shRNA cells were infected with replication-defective VSV-G pseudotyped HIV-1 for 48 h. Cell and viral lysates were separated by SDS-PAGE and probed for Gag p24 by Western blotting. The relative amount of cell-free-to-cell-associated Gag was quantified using Image J. Equal loading of cell lysates was verified by probing membranes with anti-actin antibody. A representative blot is shown with quantification from two independent experiments with the SEM is shown.

To explore the possibility that the Lyst protein mutated in CHS patients confers a block in cell-to-cell transmission and to provide a second line of evidence that the defect in HIV-1 spread in CHS cells was restricted to Lyst function, we carried out RNAi knockdown of Lyst by transducing T cells with lentivirus expressing *lyst*-specific shRNA and directly analyzed cell-to-cell spread of virus. HIV-1-infected Jurkat T cells stably expressing a pool of 4 different Lyst-shRNA sequences displayed a 25% reduction in Lyst mRNA compared to control Jurkat cells expressing scrambled shRNA sequences (Fig. S6a). Although Lyst-shRNA knockdown represented a modest reduction in Lyst mRNA levels, this was reproducible and may translate into a more extreme phenotype in reduction in protein levels. Unfortunately we are unaware of the existence of an antibody to Lyst that could be used for detection of this protein in WT and shRNA-transduced cells; however, Lyst-shRNA cells do show an altered CD63 distribution compared to control cells indicative of a defect in this pathway (Fig. S6b). To quantify cell-to-cell spread, Lyst-shRNA cells or control-shRNA expressing cells were infected with HIV-1 and incubated for 10 days to establish a chronic infection before being used as donor cells in cell-to-cell transmission assays when >90% of the cells were infected as measured by intracellular Gag staining and flow cytometry (data not shown). This allowed us to examine cell-to-cell spread to target cells while obviating spreading infection within the donor cell population. Infected donor cells were mixed with normal uninfected Jurkat target T cells for 0, 1, 3 and 6 h (a time frame that permits efficient cell-to-cell but not cell-free infection [7], [8], [44], [45], [48]) and genomic DNA was extracted and HIV-1 DNA reverse transcripts were quantified by real-time qPCR. The baseline values at t = 0 h were subtracted from each time point (to account for variation in the amount of HIV-1 provirus in infected cells) and normalized to a housekeeping gene to determine the HIV-1 DNA index, a measure of the copy number of HIV-1 DNA [7], [45]. Fig. 7a shows Lyst-shRNA cells were significantly impaired in their ability to transmit virus to target T cells by cell-to-cell spread when compared to donor cells expressing control-shRNA (6 h DNA index 12.3±2.7 compared to 42.8±12.5, *p* = 0.001).

To confirm these results using a second assay system, Jurkat cells expressing control-shRNA or Lyst-shRNA were infected with HIV-1, incubated for 3 days and mixed with uninfected 1G5 target T cells. We confirmed that very little luciferase activity within 24 h was from cell-free virus produced by donor cells, verifying that this assay efficiently detects only cell-to-cell spread (data not shown). Fig. 7b shows that in agreement with the real-time PCR results, Lyst-shRNA cells were significantly impaired in their ability to transmit infection to target 1G5 T cells by cell-to-cell spread when compared to cells expressing control-shRNA (*p*<0.001). Pre-treating target cells with the reverse transcriptase inhibitor Zidovudine reduced luciferase activity by >50% in target cells confirming that this assay detects HIV-1 infection that correlates with appropriate RT-dependent infection rather than cell-cell fusion that could drive RT-independent Tat-induced luciferase expression. Furthermore we have previously shown that most VS exist for <3 h and do not result in cell-cell fusion [45]. We used acutely-infected donor cells at 3 days post-infection when 10–30% of cells were infected, measured by intracellular Gag staining and flow cytometry analysis. Limiting the time for spreading infection within the donor population meant that equivalent numbers of infected cells were inputted into the assay enabling a direct comparison of the ability of different shRNA expressing cells to transmit virus. Moreover, because only the target cells contain the HIV-1 driven reporter construct we can unequivocally measure transmission to these cells and not ongoing spreading infection within the donor population. Using acutely-infected cells rather than chronically-infected cells as donors also demonstrated that the defect in cell-to-cell spread seen using the qPCR assay (Fig. 7a) was not dependent on the chronicity of the infection.

Finally, there was no difference in the relative infectivity of cell-free supernatants when titered on HeLa tzm-bl cells (Fig. 7c), confirming that the defect in cell-to-cell spread we observed when using Lyst-shRNA expressing cells as donor cells was not because the virus produced by these cells was non-infectious. Furthermore, when Lyst-shRNA and control-shRNA cells were infected with replication defective pseudotyped virus we did not observe any difference in Gag synthesis and release by Western blotting (Fig. 7d), showing that Lyst-shRNA expressing cells are not defective in producing cell-free virus over a single replication cycle. Taken together these data indicate that in the absence of functional Lyst protein HIV-1 infected CD4^+^ T cells are impaired in transmitting virus between T cells by cell-to-cell spread.

### The SNARE syntaxin-4 contributes to cell-to-cell spread of HIV-1 between T cells

**Figure 8:**
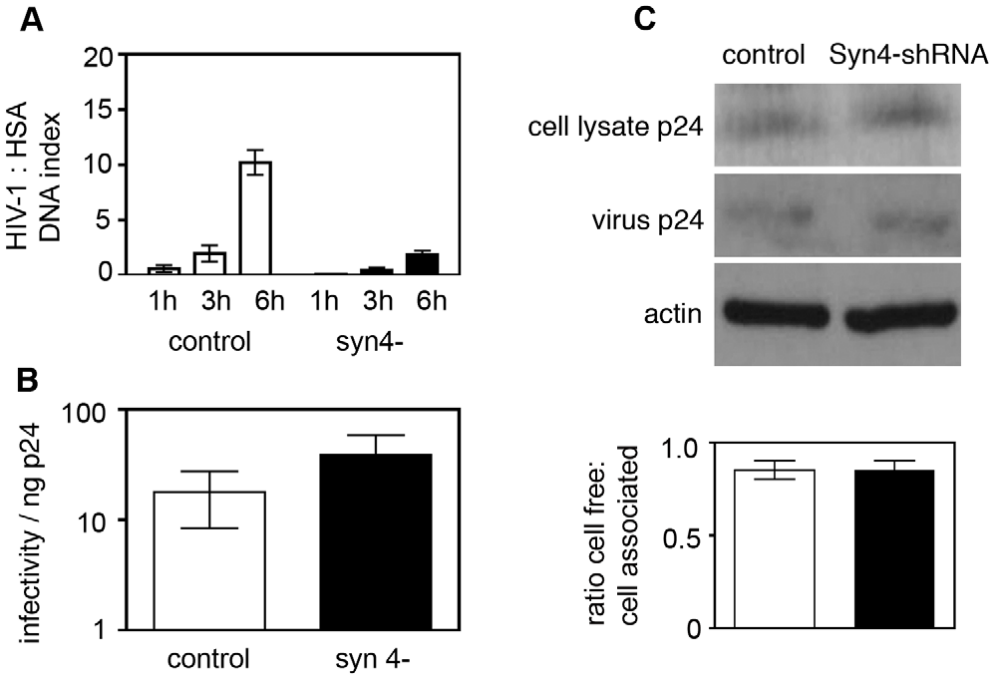
Syntaxin 4 contributes to cell-to-cell spread of HIV-1. **A**) HIV-1 infected Jurkat T cells expressing syntaxin 4-shRNA (black bars) or control-shRNA (white bars) were used to measure cell-to-cell spread of virus by quantitative real-time PCR as described in Fig. 7. Data are from three independent experiments and error bars show the SEM. **B**) *Syntaxin 4 knockdown cells produce infectious cell-free virus.* Cell-free supernatants were harvested from cells infected with replication competent HIV-1 at 7 days post-infection and viral infectivity was quantified on HeLa Tzm-bl reporter cells by luciferase assay. The TCID50 was calculated and data are shown as the TCID50 per ng of p24 to normalize for any differences in the viral content of supernatants. Data are from three independent experiments and show the SEM. **C**) *Syntaxin-4 and control knockdown cells produce equivalent amounts of p24 over a single infection cycle.* Syntaxin-4-shRNA cells and control-shRNA cells were infected with replication-defective VSV-G pseudotyped HIV-1 for 48 h. Cell and viral lysates were separated by SDS-PAGE and probed for Gag p24 by Western blotting. The relative amount of cell-free-to-cell-associated Gag was quantified using Image J. Equal loading of cell lysates was verified by probing membranes with anti-actin antibody. A representative blot is shown with quantification from two independent experiments with the SEM is shown.

To further validate a role for regulated secretion in HIV-1 VS-mediated T cell-to-T cell transmission we used lentivirus-encoded shRNA to knock down syntaxin 4, a plasma membrane-associated SNARE protein that localizes to the T cell IS and contributes to lysosome exocytosis by regulated secretion [49], [50], [51]. CD4^+^ T cells expressing syntaxin-4-specific shRNA expressed 70% less syntaxin-4 protein than control cells (Fig. S7). Similar to Lyst-shRNA expressing cells, syntaxin-4 knockdown cells were also significantly impaired in their capacity to transmit infectious HIV-1 to target T cells by cell-to-cell spread measured by qPCR (6 h DNA index 1.9±0.3, compared to 10.3±1.1, *p* = 0.001) (Fig. 8a) and by luciferase assay using 1G5 reporter T cells (Fig. 7b, *p*<0.001). Quantitative assays performed on cell-free viral supernatants harvested from Syntaxin 4-shRNA confirmed that the reduction in cell-to-cell spread was not due the production of non-infectious virus (Fig. 8b) nor were syntaxin-4 knockdown cells impaired in cell-free virus synthesis and budding when infected with single-cycle replication defective pseudovirus (Fig. 8c). We also observed that VS formation by syntaxin-4 knockdown cells was less frequent (19% ±6% of contacts evolving a VS) than cells expressing control-shRNA (68% ±2% evolving a VS *p* = 0.009). Taken together with the results using Lyst-shRNA expressing cells, these data support the concept that the cellular machinery implicated in regulated secretion from T cells contributes directly to T cell-to-T cell spread of HIV-1 at the VS.

## Discussion

Here we provide evidence that the regulated secretory pathway in CD4^+^ T cells contributes to cell-to-cell spread of HIV-1 at the VS. We have shown that HIV-1 infected T cells polarize their MTOC and associated organelles at sites of cell-cell contact that aligns the secretory apparatus proximal to the VS; that HIV-1 Env colocalizes with SL-related organelles in CD4^+^ T cells; and that CD4^+^ T cells disabled for regulated secretion are less able to support spreading HIV-1 infection. The striking polarization that we observed at the VS is reminiscent of cytoskeletal remodelling at the well-defined T cell IS where signalling induced by pMHC-TCR and integrin interactions trigger contact-mediated polarization of the MTOC and secretory apparatus for regulated secretion of vesicular cargo from T cells. At the IS, microtubules associated with the MTOC deliver secretory vesicles to the plasma membrane, a process has been elegantly imaged in CTLs [23], [24]. Although the MTOC in the HIV-1 infected T cell was frequently oriented towards the VS we rarely observed very close membrane apposition of the centrosome with the plasma membrane, rather the MTOC was located on average 1 µm from the plasma membrane at the VS. This is in broad agreement with calculations made at the IS in which very close apposition of the MTOC at the plasma membrane was not the predominant phenotype but was seen in approximately 17% of CTL synapses examined [23], and in most cases the MTOC was polarized towards the contact site but distal from the secretory domain at the plasma membrane [23]. Partial polarization (without obvious plasma membrane docking) of the MTOC at both the IS and the VS might be explained by oscillation of the MTOC at sites of cell-cell contact (to facilitate continual scanning of the synapse) [52] and/or the effect of chemical fixation only revealing a snapshot of dynamic processes. Alternatively, actual docking of the MTOC at the VS may not be essential for HIV-1 cell-to-cell spread. For example, it has been proposed that secretory compartments may be delivered to synapses either by short microtubules that contact the plasma membrane (presumably requiring close MTOC-PM apposition), via longer microtubules that curve past the synapse or by granules that dock at the edge of the synapse and move tangentially before fusing at the plasma membrane [23], [53]. In any (or all) of these ways, MTOC polarization near to the contact site may help deliver viral and/or cellular proteins to the VS by microtubule-based vesicular movement [7] and MTOC-driven organelle recruitment.

Concomitant with cytoskeletal remodelling at the VS we observed striking polarization of organelles diagnostic for regulated secretion at the IS [25], [29], [54], [55]. Notably, this polarization was only seen within the HIV-1 infected T cell and was associated predominantly with cell-cell contact, suggesting that polarization may be driven by interactions at the HIV-1 T cell VS. The T cell IS (particularly the cytotoxic IS) is characterized by a broad, integrin-rich peripheral ring with a central secretory domain that serves as a focal point for regulated secretion. By contrast, our EM tomography of the HIV-1 VS and other studies [2], [9], [45] reveals an interface that is structurally similar but with smaller, numerous areas of cell-cell contact that is accessible to soluble proteins [45]. Planar bilayer studies have elegantly demonstrated actin clearing and molecular segregation at the T cell VS reminiscent of the p- and cSMACs seen at the IS [56], [57]. Therefore whilst similarities between the ultrastructure of the IS and the T cell VS exist, there are also clear differences that probably reflect the effects of different receptor-ligand interactions.

Collectively, our data generated using WT and CHS patient-derived cells and RNAi suggests that cell-to-cell spread of HIV-1 at the VS may functionally intersect the T cell regulated secretory pathway. Hijacking this machinery and T cell polarization would permit HIV-1 exquisite temporal and spatial control over virus assembly and exit, both by polarizing dissemination at the VS, as we have previously reported [2], and potentially by limiting the release of cell-free particles until the brief window of time in which the infected cell contacts an uninfected, receptor-expressing target cell. Based on the data presented here, we hypothesize that the activation of a contact-dependent secretory pathway may contribute to directing retroviral proteins to intercellular junctions. Polarization of HIV-1 assembly and budding at sites of cell-cell contact has been described [2], [4], [8], [9], [58], [59], [60], [61], [62], [63], [64] but the cellular processes underlying this are ill-defined. Similarly, preferential assembly of the related retrovirus MLV at sites of cell-cell contact has also been reported but again the underlying mechanism for this is unknown [65]. At the T cell IS polarization and activation of regulated secretion is triggered by T cell receptor engagement with degranulation and secretion determined by the strength of TCR signal [28], [53]. In addition, adhesion molecules such as the LFA-1 and ICAM play a central role alongside pMHC-TCR in triggering polarization and regulating secretion at IS [66], [67], [68], [69]. The TCR is not specifically enriched at the HIV-1 T cell VS and homotypic T cell-T cell interactions and the T cell VS are antigen-independent structures. VS assembly is driven by Env-receptor and LFA-1-ICAM interactions [2], [70] and we hypothesize a model in which small amounts of Env present at the plasma membrane may initially engage viral receptors on the target T cell and together with integrins help provide a stable adhesive environment, recruit and activate additional adhesion molecules and potentially signal into the infected cell to polarize elements of the secretory apparatus proximal to the VS, as demonstrated for HTLV-1 and implied by studies using immortalized HIV-1 infected T cell lines [7], [71], [72]. This in turn could help deliver viral and cellular proteins via the regulated secretory pathway to the plasma membrane at the VS.

HIV-1 Env mediates attachment to, and entry into susceptible cells, directs the site of virus budding at the plasma membrane, and drives VS formation leading to cell-to-cell viral spread; however, little is known however about how Env is directed to the sites of HIV-1 assembly at the plasma membrane, and by extension to the VS. Here we show that CD4^+^ T cells contain a functional SLRO in which HIV-1 Env and SL-associated proteins colocalize both intracellularly and at the VS suggesting that the pathways of regulated secretion and Env trafficking may intersect. We report that two proteins trafficked by regulated secretion in T cells, CTLA-4 and FasL are also enriched along with Env at the VS. Colocalization of HIV-1 and CD63 at sites of virus budding and the VS has been observed previously by ourselves and others suggesting that the pathway of HIV-1 egress and vesicular trafficking may overlap [31], [73], [74], [75]. Env is normally trafficked to the surface of infected cells but is endocytosed via association with clathrin adaptor proteins [36], [37], [38], [40], [41], [42], [46], [76]. This is similar to the transport pathways employed by CTLA-4 that can access SL-related compartments either by trafficking directly from the Golgi or after passage via the plasma membrane and association with clathrin adaptor complexes [16], [17], [77]. We detected the Env subunit gp120 on the surface of WT and CHS CD4^+^ T cells infected with HIV-1, providing evidence for functional constitutive transport. However, Env appeared to accumulate in SLRO in CHS T cells following endocytosis from the plasma membrane suggesting that HIV-1 Env may initially traffic to the plasma membrane in infected T cells, from where it gets endocytosed ending up in lysosome-like compartments. The colocalization we observed between Env and SLRO, coupled with the polarization of secretory apparatus and the MTOC within the infected T cell towards the VS during cell-cell contact, and the defect in cell-to-cell spread when regulated secretion was disabled may be explained by cell-to-cell spread at the VS intersecting the regulated secretory pathway in T cells. In cells defective for regulated secretion (e.g. CHS cells) Env might accumulate in immobile secretion-defective SLRO and/or be incorrectly sorted to other compartments. In this way, Env may not be effectively recycled back to the plasma membrane or mobilized to the VS following stimulation of regulated secretion. Based on our data, we cannot exclude the possibility that the defect in regulated secretion may also be affecting cell-to-cell spread of HIV-1 in an Env-independent manner and that regulated secretion at the T cell VS may similarly control the delivery or endocytosis of other proteins (for example adhesion molecules) that in turn could affect the frequency or duration of cell-cell contacts. Moreover, although HIV-1 Gag did not colocalize with Env in SLRO, it is possible that activation of regulated secretion during cell-cell contact may also direct Gag to VS but via an alternate transport pathway [9], [64], [65].

In polarized T cells HIV-1 Gag localizes to uropods and this is suggested to facilitate cell-to-cell transmission of virus [78]. Uropods are cellular projections rich in adhesion molecules and organelles and are found in activated T cells that have established front-rear polarity (e.g. during chemotaxis). Notably, Gag enrichment in uropods was reported to be Env-independent [78], and it was suggested that Env may not be required for initial contact via integrin-rich uropods, presumably because a critical concentration of adhesion molecules were already present. By contrast, Env-receptor interactions have been shown by us and others to be necessary for stable T cell-T cell contacts, VS formation and transmission of infectious HIV-1 by cell-to-cell spread [2], [4], [8], [9], [45]. Our EM tomography and confocal microscopy data show that cell-cell contact is associated with striking organelle and cytoskeletal polarization within the HIV-1 infected T cell that correlates with virus budding at the VS. T cell polarization at sites of virus budding was much less evident in unconjugated T cells, suggestive of contact-induced rearrangements. Thus it appears that T cell contacts leading to VS formation and HIV-1 transmission may be initiated by (at least) two mechanisms: In the first model supported by the data presented here, contact between an HIV-1 infected T cell and a target T cell may trigger cytoskeletal remodelling and enrichment of the MTOC and secretory organelles towards the contact site establishing a polarized phenotype within the HIV-1 infected T cell. In this situation, the initial contact could be mediated by Env-CD4 and/or LFA-1-ICAM interactions with further recruitment of additional Env and integrins to stabilize the contact site and establish the VS. The induction of a polarized phenotype could then help focus Env secretion via activation of a regulated transport pathway for efficient virus assembly and cell-to-cell spread. In the second model supported by the data of Llewellyn et al., enriched integrins at uropods in pre-polarized HIV-1 infected cells could themselves initiate contact between HIV-1 infected T cells and target T cells and stabilize conjugate interfaces. Because the MTOC and organelles would be already located at the uropod in such polarized T cells the secretory apparatus could be poised to contribute to Env trafficking towards the plasma membrane at the contact site for infectious virus assembly. It should be noted that these models are not mutually exclusive and it is possible that different processes may operate under different conditions depending on whether a T cell is polarized before or during cell-cell contact. Future work will seek to determine what triggers contact-dependent polarization at the T cell VS and how this may contribute to activation of secretion and HIV-1 protein trafficking to sites of cell-cell contact. Live cell imaging of Env trafficking to VS would shed much light on this area but such studies will be considerably more challenging.

The cellular protein Lyst is thought to function in the secretory pathway by regulating membrane fusion/fission, and defects in lysosome morphology may be influenced by interactions between Lyst and members of the ESCRT (endosomal sorting complex required for transport) pathway, a group of cellular proteins that normally regulate vesiculation and cytokinesis but also promote HIV-1 budding at the plasma membrane [79]. Here we provide new evidence that Lyst may also be involved in regulating cell-to-cell spread of HIV-1, perhaps by modulating Env trafficking. It is worth noting that the ESCRT machinery and MVB-associated lipids are also recruited to the IS [80], [81] and MVB-like structures related to SL form at the CD4^+^ T cell IS where TSG101 recognition of ubiquitinated protein is necessary for TCR downregulation and cSMAC formation [80]. In the context of HIV-1 egress, TSG101 is recruited to sites of virus budding by association with late-domain motifs in Gag and is necessary to mediate the release nascent HIV-1 particles from the plasma membrane of infected cells. Whether ESCRT helps regulate cell-to-cell spread of HIV-1 at the T cell VS by other as yet undefined mechanisms is an intriguing possibility that warrants further investigation. Within cells the delivery of vesicular cargo is controlled by the SNARE protein family - a group of proteins that promote fusion between vesicles and target cell membranes through specific t-SNARE and v-SNARE interactions. The SNARE proteins syntaxin 4 is implicated in regulated secretion from haematopoetic cells and at the IS [49], [50], [82], [83] and we have demonstrated that syntaxin 4 also contributes to HIV-1 egress at the VS. Deregulating SL function via natural Lyst mutation, or with RNAi, and inhibition of syntaxin 4 mediated vesicle fusion identified these two proteins as molecules that contribute to HIV-1 spread at the VS. Interestingly, secretion of soluble proteins by CD4^+^ T cells at the IS requires different cellular machinery depending whether proteins are secreted in a polarized or multidirectional manner [22] and this may be true also for HIV-1 egress at the VS since we saw no effect of Lyst or syntaxin 4 on cell-free HIV-1 release but observed a clear defect in cell-to-cell spread. Future studies will aim to uncover other proteins that are involved in the pathway of HIV-1 egress at the VS. Delineating the pathway of cell-to-cell spread of HIV-1 and the immunological consequences thereof may identify novel targets for rational drug-design that can specifically inhibit this mode of HIV-1 egress and limit HIV-1 pathogenesis.

## Materials and Methods

### Cells and viruses

Peripheral blood mononuclear cells were separated by density gradient centrifugation (Histopaque, Sigma) and CD4^+^ T cells were purified by negative magnetic selection (Miltenyi Biotech). Two clonal IL-2 dependent CD4^+^ T cell lines derived from a patient with Chediak-Higashi Syndrome were generously provided by Prof. G. Griffiths (The University of Cambridge, United Kingdom) [34]. Dr Alain Fischer and Dr Genevieve de Saint Basile (Hopital Necker Enfants Malades and INSERM Paris) also kindly provided PBMCs from second CHS patient from which we purified CD4^+^ T cell PHA blasts after *in vitro* stimulation with 1 µg/ml PHA (Sigma) and 10 IU/ml IL-2 (Center for AIDS Reagents [CFAR], National Institute for Biological Standards and Control [NIBSC], UK). When initially isolating CD4^+^ T cells from CHS patient PBMCs, WT PBMCs were obtained from the peripheral blood normal healthy donors by gradient centrifugation of buffy coats (Brentwood Blood Center, National Health Service, UK), cultured in parallel with CHS cells and CD4^+^ T cells were simultaneously purified. Control CD4^+^ T cells used were either CD4^+^ T cell clones or CD4^+^ T PHA blasts purified from activated PBMCs obtained from at least four normal healthy donors. Primary cells were maintained in RPMI 1640 (Invitrogen) containing 5% human serum, 0.75% sodium bicarbonate (Invitrogen), 1 mM sodium pyruvate (Invitrogen), 2 mM L-glutamine (Invitrogen), 50 µM mercaptoethanol (Sigma) and 10 IU/ml IL-2. To allow direct comparison in infection studies all CHS and control CD4^+^ T cells were maintained *in vitro* by synchronous restimulation every 21 days with irradiated allogeneic peripheral blood mononuclear cells and 1 µg/ml PHA. Cells were also cryogenically preserved for long-term storage to limit repeated stimulations. The CD4^+^/CXCR4^+^ T cell line Jurkat CE6.1 (American Type Culture Collection) and the derivative Jurkat line 1G5 containing the firefly luciferase gene driven by the HIV-1 LTR (obtained through the AIDS Research and Reference Reagent Program, Division of AIDS, NIAID, NIH: from Dr. Estuardo Aguilar-Cordova and Dr. John Belmont)[43] were maintained in RPMI 1640 supplemented with streptomycin (100 µg/ml), penicillin (100 U/ml) and 10% fetal calf serum (FCS, Invitrogen). For infection of primary cells with HIV-1, surface expression of CD4, CXCR4 and CCR5 was measured by flow cytometry and cells infected with either the CXCR4-tropic strain IIIB or the CCR5-tropic strain BaL as described [7]. Jurkat cells were infected with CXCR4-tropic strain IIIB. HIV-1 infected cells were phenotyped as described [7].

### Lentiviral shRNA knockdown in Jurkat T cells

Jurkat CE6.1 cells were infected with a Mission® shRNA lentiviral transduction particle set targeting Lyst (clones NM_000081.1-3696s1c1, NM_000081.1-10178s1c1, NM_000081.1-493s1c1, NM_000081.1-5055s1c1 and NM_000081.1-2538s1c1), syntaxin 4 (NM_004604.3-574s1c1, NM_004604.3-360s1c1, NM_004604.3-646s1c1, NM_004604.3-855s1c1, NM_004604.3-881s1c1) or lentivirus encoding a scrambled shRNA control (clone SHC002V) according to manufacturer's instructions (Sigma). Stable, lentivirus infected cells (designated Lyst-shRNA, syntaxin 4-shRNA and control-shRNA) were selected with puromycin (Sigma) and maintained in RPMI 1640 supplemented with 10% FCS, streptomycin (100 µg/ml), penicillin (100 U/ml) and puromycin. Lyst mRNA levels were quantified with reference to GAPDH using an Applied Biosystems 7500 Fast Real-Time PCR System and Quantitect Primer and Sybr Green Probe Assay kits (Qiagen). Syntaxin 4 knockdown was quantified by Western blotting of cell lysates prepared from untreated Jurkat cells and shRNA expressing cells using a mAb against sytaxin 4 (clone 49, BD Biosciences) and a rabbit anti-actin serum (Sigma) as described previously [7].

### Flow cytometry

CD4^+^ T cells were washed in cold fluorescence-activated cell sorter (FACS) wash buffer (FWB: PBS with 1% FCS and 0.01% sodium azide) and incubated for an hour on ice with mAbs against CD4 (clone Q4120, donated by Q Sattentau and obtained from the CFAR), CXCR4 (clone 12G5, donated by J Hoxie and obtained from the CFAR), CCR5 (clone 2D7, obtained through the AIDS Research and Reference Reagent Program, Division of AIDS, NIAID, NIH) or HIV-1 Env (IgG1b12, donated by D. Burton and P Parren and obtained from the CFAR; 2G12 from Polymun, Vienna). Cells were washed and incubated with anti-mouse or anti-human IgG-phycoerythrin for 30 min on ice, fixed with 1% formaldehyde and analyzed using a Becton Dickinson FACS Calibur and CellQuest Software. Intracellular Gag staining was performed by fixing cells, permeabilizing in perm/wash buffer (BD Biosciences) and incubating with the Gag-specific mAb KC57-PE (Beckman Coulter). For degranulation assays with uninfected T cells, CD4^+^ T cells were either left untreated or incubated with 50 ng/ml PHA (Sigma) and 1 µg/ml ionomycin (Sigma) for up to 1 h at 37°C in the presence of antibody against CTLA-4 (mAb BNI3, BD Biosciences) or CD63 (a gift from G. Griffiths and obtained from the Developmental Studies Hybridoma Cell Bank, University of Iowa). Cells were washed, stained with PE-conjugated anti-mouse secondary antibody and analyzed by flow cytometry.

### Immunofluorescence laser scanning confocal microscopy

CD4^+^ T cells were washed in RPMI 1640-1% FCS (WB) and incubated on poly-L-lysine (Sigma)-treated coverslips at 37°C for 30–60 minutes. In some cases, T cells were first activated on immobilized anti-CD3 (1/1000 UCHT1 and 10 µg/ml OKT3) for 24 hr, detached with cold PBS-5 mM EDTA, washed extensively and resuspended in WB. Coverslip-adhered cells were fixed in 4% formaldehyde for 15 minutes, permeabilized in 0.1% Triton X-100-5% FCS or cold 100% methanol and washed extensively in PBS-1% BSA. Immunostaining was performed with the following antibodies: CTLA-4 was detected with the mouse mAb BNI3 (IgG2_a_, BD Biosciences); FasL with NOK-1 (IgG_1_, BD Biosciences); cathepsin D with polyclonal rabbit antibody (Upstate); CD63 with mAb IB5 (IgG2_b_ provided by M.Marsh, University College London, UK); mitochondrial protein ATP synthase subunit β with mAb 3D5 (IgG1, Abcam. A gift from M. Duchen, University College London); HIV-1 Env with the human mAb 2G12 (Polymun Scientific) or 50–69 (donated by S. Zoller-Pasner and obtained from the CFAR) and HIV-1 Gag with rabbit antisera against p17 and p24 (donated by G. Reid and obtained from the CFAR) or the mouse mAb clone EH12E1 (donated by R. Ferns and obtained from the CFAR). Primary antibodies were detected with anti-human or anti-rabbit FITC, TRITC or Cy5-conjugated secondary antibodies (Jackson Immunoresearch) and isotype-specific anti-mouse Alexa-488, Alexa-568 and Alexa-647 (Invitrogen). Coverslips were mounted with ProLong antifade (Invitrogen) and images were acquired using either a BioRad Radiance 2000 MP or a Leica TCP SP2 laser scanning confocal microscope with excitation wavelengths of 488, 568 or 633 nm using 63x and 40x oil immersion objectives (NA 1.4). Image processing was performed using Metamorph v.7 (Molecular Devices) and Photoshop v.9 (Adobe). Colocalization was quantified using Metamorph, single *xy* slices were extracted from the middle section of multiple cells (>20 per staining), thresholded and correlation plots were generated to determine the Pearson's correlation coefficient (r).

To visualize endocytosed HIV-1 Env in CHS and WT cells, T cells were infected with either BaL or NL43 at an MOI of 0.01 for 4 days, washed and incubated in fresh media supplemented with 10 µg/ml of the CD4 binding-site specific anti-Env mAb IgG1b12 for 24 h at 37°C. Cells were washed, fixed, permeabilized, and stained with anti-CD63 or anti-CTLA4 primary antibodies followed by anti-human secondary antibody (to detect IgGb12) and anti-mouse isotype specific secondary antibodies (to detect CD63 and CTLA-4) and examined by LSCM.

For conjugate and synapse experiments HIV-1 infected CHS or WT CD4^+^ T cells were mixed with autologous uninfected CD4^+^ T cells and incubated on coverslips in the presence of the non-blocking HIV-1 Env mAb 50–69 for up to 2 h at 37°C as described previously [2]. Conjugates were then fixed, permeabilized and stained for HIV-1 Gag or SL-associated proteins as described above. Alternatively, HIV-1 infected T cells were mixed with autologous target T cells prestained with the anti-CD4 mAb L120 (donated by D Healey and obtained from the CFAR) and incubated for 1 h at 37°C before being fixed, permeabilized and stained for γ-tubulin (Sigma) and HIV-1 Env with the mAb 2G12. Primary antibodies were detected with species and isotype-specfic antibodies and analyzed by LSCM as described above. For quantification conjugates were defined as two closely apposed cells consisting of one CD4^+^ target cells and one HIV-1 infected cell (Env^+^ or Gag^+^), a VS was defined as a conjugate showing enrichment of viral proteins to sites of cell-cell contact.

### Electron microscopy and tomography

HIV-1 infected Jurkat T cells were mixed with target CD4^+^ T cells and prepared for electron microscopy as described previously [2]. 70 nm and 300 nm serial sections were obtained with a Leica Ultracut UCT microtome and a diamond knife. Cell morphology was assessed and quantified in 70 nm sections of two independent samples and on two grids per sample in a systematic random manner with a Philips Morgnani 268 TEM (100 kV). 2D images were taken with a CCD camera on the same TEM. Tomography was carried out essentially as described elsewhere [84]. Single or dual axis digital image series from 300 nm sections were recorded on a TECNAI microscope operated at 300 kV (4k FEI Eagle camera at a pixel size of 1.99 nm, 2.54 nm or 4.96 nm on the specimen level) over a −60° to 60° tilt range (increment 1°) and at a defocus of −0.2 µm. Tomograms were reconstructed and joined using the IMOD software package (version 4.1.4)[85]. The AMIRA Visualization Package (version 5.2.0 Visage Imaging, Berlin, Germany) was used for 3D image segmentation of the 2x binned volumes by manually masking areas of interest, mask thresholding and label smoothing. The volumes were Gauss filtered (2×2×2 kernel) for presentation in Videos S1–S4.

### Quantification of HIV-1 replication in CHS and WT CD4^+^ T cells

For infection studies up to 5×10^6^ CHS cells and WT cells were infected in parallel with the HIV-1 strain BaL or IIIB at an MOI of 0.005. To quantify HIV-1 production in cultures infected with replication competent virus (BaL or IIIB), cell-free supernatants were collected from infected cells on days 4, 7 9 and 12 post-infection and HIV-1 release was quantified by Gag p24 ELISA [7]. Viral infectivity of cell-free supernatants was measured on HeLa Tzm-bl reporter cells (donated by J. Kappes, X. Wu and Tranzyme Inc. and obtained from the Center for AIDS Reagents, National Institutes of Biological Standard and Control, UK) using the Bright-Glo Luciferase assay kit (Promega). Viral infectivity was normalized to Gag p24 to calculate the TCID_50_/ng p24 to account for variation on HIV-1 release. To examine HIV-1 Env incorporation into budding virions, virus was purified from cell-free supernatants harvested from infected CHS and WT cultures by ultracentrifugation through a 25% sucrose cushion, resuspended in PBS and stored at −80°C. Equal volumes of virus in sample buffer were separated by SDS-PAGE on a 4–12% Bis-Tris gradient gel (Invitrogen), transferred to nitrocellulose for Western blotting and probed with rabbit antiserum raised against HIV-1 gp120 (donated by S.Ranjbar and obtained from the CFAR) and HIV-1 Gag (donated by G. Reid and obtained from the CFAR) followed by goat anti-rabbit-HRP and visualized by enhanced chemiluminescence (ECL) (Amersham).

### Single cycle virus release assay

293T cells were co-transfected with the VSV-G expressing plasmid pMDG [86] and the Env- plasmid pNL4-3Luc.R.E (obtained through the NIH AIDS Research and Reference Reagent Program, Division of AIDS, NIAID, NIH: from Dr. Nathaniel Landau)[87] using PEI (Sigma) and VSV-G pseudotyped, replication-defective HIV-1 was harvested after 48 h and quantified on HeLa Tzm-bl cells and by p24 ELISA. For single-cycle virus release assays 1×10^6^ WT or CHS CD4^+^ T cells were infected with 100 ng pseudovirus for 16 h, washed to remove excess virus and incubated in fresh media for 48 h. Cell-free supernatants containing Gag p24 from the single-cycle infection were harvested and the cell pellets were lysed with RIPA buffer (radioimmunoprecipitation buffer: 50 mM Tris-HCl [pH 8], 150 mM NaCl, 1% NP-40, 0.1% SDS, 0.5% deoxycholic acid, 1 mM phenylmethlysulfonyl fluoride [PMSF], and Complete protease inhibitor cocktail [Roche, UK]) on ice for 10 min and soluble protein was collected by centrifugation at 15,000× *g* for 10 min at 4°C. Protein concentrations were determined using the BCA Protein Assay Reagent Kit (Pierce). Twenty micrograms of cell lysate and an equal volume of virus in sample buffer were separated by 10% SDS-PAGE, transferred to nitrocellulose for Western blotting and probed with rabbit antiserum raised against HIV-1 Gag and anti-actin (Sigma) followed by goat anti-rabbit-HRP and visualized by ECL.

### Quantification of HIV-1 entry into WT and CHS cells

1×10^6^ CD4^+^ primary T cells were infected with the VSV-G pseudotyped pNL4-3 Luc.R-E- virus at an MOI of 0.01 for 4 h, excess virus was removed by washing and cells were incubated in media for 24 h at 37°C. The cells were subsequently lysed and infection was quantified by measuring luminescence using the Bright-Glo luciferase assay (Promega).

### Quantification of HIV-1 T cell-T cell spread

5×10^5^ Lyst-shRNA, syntaxin 4-shRNA or control-shRNA cells chronically infected with HIV-1 IIIB at an MOI of 0.005 (10 days post-infection) were mixed with an equal number of uninfected T cells for up to 6 hours at 37°C, pelleted by centrifugation and the genomic DNA extracted. Quantitative real-time PCR was performed using an ABI 7000 to detect HIV-1 reverse transcripts as a measure of cell-to-cell spread as described previously [7], [45]. The HIV-1 *pol* copy number was normalized to the *albumin* or *β-globin* housekeeping gene and the baseline signal at time point 0 h was subtracted to account for the starting level of infection. Alternatively, Lyst-shRNA, syntaxin 4-shRNA or control-shRNA cells were infected with the HIV-1 strain IIIB at an MOI of 0.01, incubated for 3 days and 1×10^5^ cells donor cells were mixed with an equal number of 1G5 Jurkat T cells and incubated for 24 h at 37°C. 1G5 target cells were either left untreated or pretreated for 1 h at 37°C with the reverse transcriptase inhibitor Zidovudine to achieve a final concentration of 50 µM (obtained from the NIH AIDS Research and Reference Reagent Program) prior to mixing with donor cells. After 24 h the cells were washed, lysed and luciferase activity was measured by luminescence using the Bright-Glo luciferase assay. To quantify the percentage of HIV-1 infected donor cells 2×10^5^ cells were fixed in 4% formaldehyde, washed, permeabilized and stained with the PE-conjugated HIV-1 Gag specific antibody and analyzed by flow cytometry using a FACSCalibur. The percentage of infected cells was quantified with reference to uninfected Jurkat cells stained for Gag. To quantify cell-to-cell spread with WT and CHS T cells, cells were infected with the HIV-1 strain NL4-3 at an MOI of 0.01, incubated for 48 h and 1×10^5^ cells donor cells were mixed in triplicate with an equal number of 1G5 Jurkat T cells and incubated for 24 h at 37°C. After 24 h the cells were washed, lysed and luciferase activity was measured by luminescence using the Bright-Glo luciferase assay.

### Statistical analysis

Statistical significance was calculated either using the parametric Anova test for multiple comparisons with Bonferroni correction, or the non-parametric Mann-Whitney test. Significance was assumed when *p*<0.05. Pearson's correlation coefficients (*r* values) were calculated using Metamorph.

## Supporting Information

Figure S1 **Unpolarized morphology of uninfected and unconjugated T cells.** Infected Jurkat T cells and primary CD4^+^ T cells were fixed and embedded. a) – c) 70nm thin sections were post-stained with lead citrate and examined in the electron microscope. a) Conjugate of two uninfected cells, both unpolarized. b), c) single HIV-1 infected (b) and uninfected (c) cells, both unpolarized as seen by the even distribution of cellular organelles around the nucleus (outlined in red). d) – f) single computational slices and 3D surface rendering of reconstructed tomograms from 300nm sections. Cells are unpolarised, as seen by the even distribution of secretory organelles (endoplasmic reticulum, ER, not pseudocolored), secretory lysosomes (SL, orange), Golgi (not pseudocolored), mitochondria (m, green) and lipid bodies (LB, pale blue) around the nucleus (brown). Arrows in b) and e) indicate virus particles (red) released from infected cells (plasma membrane depicted in red). Scale bars =  1 µm.
(TIF)

Figure S2 **Localization of cellular proteins to secretory lysosomes in CD4^+^ T cells**. **A**) CD4^+^ T cells were purified from healthy donors, activated, permeabilized and stained for CD63, cathepsin D, CTLA-4 and FasL. Primary antibodies were detected with fluorophore-conjugated anti-rabbit and isotype-specific anti-mouse secondary antibodies. Images are 3D reconstructions of serial *z-*sections taken by LSCM with the corresponding merged and DIC images are shown. Scale bar  =  5μm. **B**) Quantification of colocalization (average r value) between cathepsin D and CD63 (n = 31), cathepsin D and CTLA-4 (n = 21), cathepsin D and FasL (n = 20), CD63 and CTLA-4 (n = 20), and CD63 and FasL (n = 20) is shown. Quantification was performed using single *xy* slices. Error bars are the SEM from four experiments performed using six individual donors.
(TIF)

Figure S3 **CD3 antibody is not internalized following T cell activation**. CD4^+^ T cells were activated on plate-immobilized anti-CD3, detached with EDTA, washed, permeabilized and stained with isotype-specific anti-mouse IgG1 (top panel), IgG2a (middle) and IgG2b (lower) secondary antibody and analysed by LSCM. We did not detect intracellular CD3 staining, confirming the specificity of the CD63, CTLA-4 and FasL staining.
(TIF)

Figure S4 **CHS CD4^+^ T cells show a defect in degranulation.** Normal CD4^+^ T cells (white bars) or CHS CD4^+^ T cells (black bars) were either untreated (UT) or stimulated with PMA-ionomycin for 10 min or 60 min in the presence of mAbs specific for CTLA-4 or CD63. Cells were washed, stained with anti-mouse phycoerythrin-conjugated secondary antibody and surface expression of CTLA-4 (top panel) and CD63 (lower panel) was measured by flow cytometry. Graphs show the MFI and SEM of data combined from four experiments performed with four independent WT donors and two different CHS clones.
(TIF)

Figure S5 **Gag does not localize to SL-related compartments in CHS CD4^+^ T cells**. HIV-1-infected CHS cells were fixed, permeabilized and stained for Env (red), Gag (blue) and CD63 (green). Images are 3D reconstructed *z*-series and areas of colocalization are yellow.
(TIF)

Figure S6 **Validation of Lyst knockdown in Jurkat T cells.** **A**) Total cellular RNA was extracted from Jurkat T cells stably expressing scrambled shRNA sequences (control cells, white bars) or Lyst-specific shRNA (black bars) and the relative quantification of Lyst mRNA determined by real-time reverse transcription PCR. Data are expressed as relative mRNA levels compared to control cells that are normalized to 1. Error bars represent the SEM from multiple experiments. **B**) Cells expressing scrambled shRNA (top panel) or Lyst-shRNA (bottom panel) were fixed, permeabilized and stained for CD63 (green).
(TIF)

Figure S7 **Quantification of syntaxin 4 knockdown in Jurkat T cells.** Total cell lysates were separated by SDS-PAGE and Western blotting performed to determine the relative expression of syntaxin 4 protein in control-shRNA expressing cells, syntaxin 4-shRNA expressing cells and untreated Jurkat cells. The relative quantification of sytaxin 4 levels determined by densitometer analysis is shown.
(TIF)

Video S1 **Polarization of HIV-1 infected T cells at the virological synapse.** Animation through a z-series of 3.99nm thick digital slices of a dual axis tomogram, reconstructed from two sequential 300nm thick sections of Jurkat cells mixed with uninfected CD4^+^ T cells. Colored overlay is a 3D surface model of organelle redistribution towards the site of virus particle (red) release in the infected cell. Plasma membranes (semi-transparent) are depicted in red (infected cell) and yellow (uninfected cell) and the nucleus in brown. Organelles are rendered as follows: mitochondria (green), lipid bodies (pale blue), lysosomes (orange) and microtubule organizing centre (blue).
(MOV)

Video S2 **Uninfected T cells conjugates are non-polarized.** Animation through a z-series of 5nm thick digital slices of a single axis 1x2 montage tomogram, reconstructed from a 300nm thick section of Jurkat cells mixed with uninfected CD4^+^ T cells. Colored overlay is a 3D surface model of the unpolarized morphology. Plasma membranes (semi-transparent) are depicted in yellow and the nucleus in brown. Organelles are rendered as follows: mitochondria (green), lipid bodies (pale blue) and lysosomes (orange).
(MOV)

Video S3 **Unconjugated, infected T cells are non-polarized.** Animation through a z-series of 5nm thick digital slices of a single axis tomogram, reconstructed from a 300nm thick section of a single, infected Jurkat T cell is shown. Colored overlay is a 3D surface model of the unpolarized morphology. Plasma membrane (semi-transparent) is depicted in red and the nucleus in brown. Organelles are rendered as follows: mitochondria (green), lipid bodies (pale blue) and lysosomes (orange). Virus is colored red.
(MOV)

Video S4 **Unconjugated, uninfected T cells are non-polarized.** Animation through a z-series of 2.5nm thick digital slices of a single axis tomogram, reconstructed from a 300nm thick section of a single, noninfected T cell is shown. Colored overlay is a 3D surface model of the unpolarised morphology of the two uninfected cells. Plasma membrane (semi-transparent) is depicted in yellow and the nucleus in brown. Organelles are rendered as follows: mitochondria (green), lysosomes (orange).
(MOV)
