# Supplementary figures and images for: The Regulated Secretory Pathway in CD4+ T cells Contributes to Human Immunodeficiency Virus Type-1 Cell-to-Cell Spread at the Virological Synapse
Source: PLoS Pathog. 2011 Sep 1;7(9):e1002226. doi: 10.1371/journal.ppat.1002226 (PMC3164651; doi:10.1371/journal.ppat.1002226)

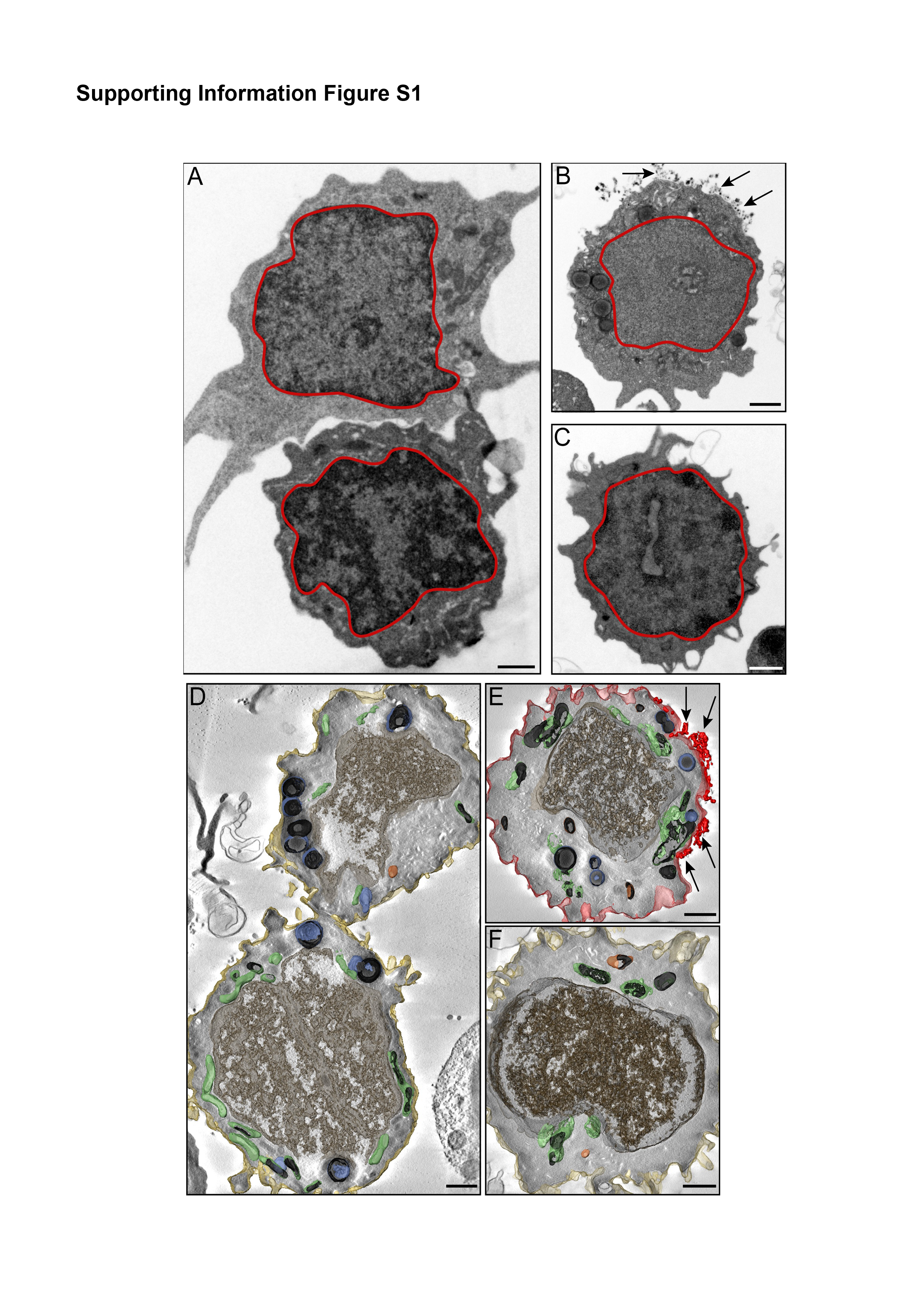

Supplement: Figure S1 — Unpolarized morphology of uninfected and unconjugated T cells. Infected Jurkat T cells and primary CD4+ T cells were fixed and embedded. a) – c) 70nm thin sections were post-stained with lead citrate and examined in the electron microscope. a) Conjugate of two uninfected cells, both unpolarized. b), c) single HIV-1 infected (b) and uninfected (c) cells, both unpolarized as seen by the even distribution of cellular organelles around the nucleus (outlined in red). d) – f) single computational slices and 3D surface rendering of reconstructed tomograms from 300nm sections. Cells are unpolarised, as seen by the even distribution of secretory organelles (endoplasmic reticulum, ER, not pseudocolored), secretory lysosomes (SL, orange), Golgi (not pseudocolored), mitochondria (m, green) and lipid bodies (LB, pale blue) around the nucleus (brown). Arrows in b) and e) indicate virus particles (red) released from infected cells (plasma membrane depicted in red). Scale bars = 1 µm. (TIF) [file ppat.1002226.s001.tif]

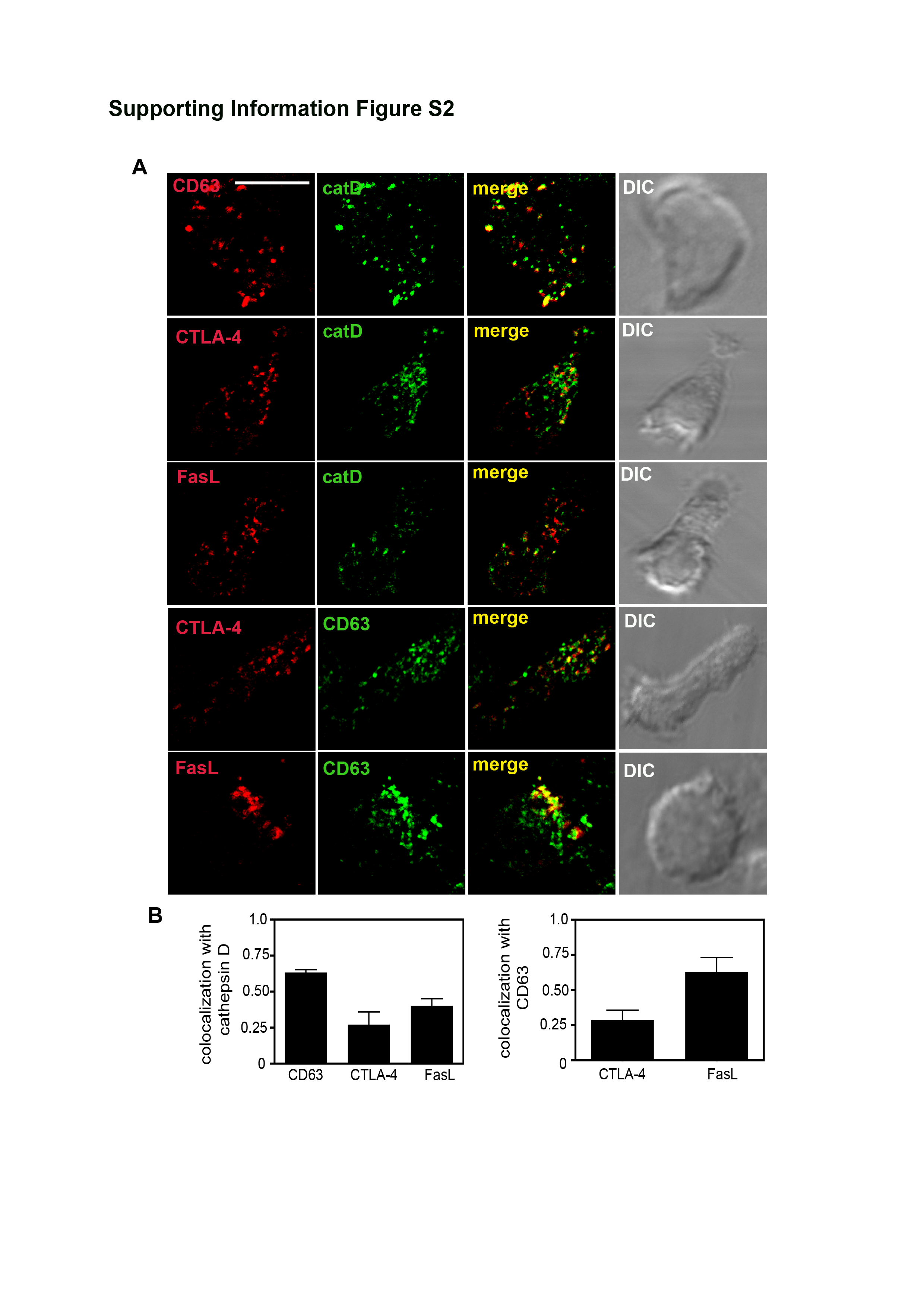

Supplement: Figure S2 — Localization of cellular proteins to secretory lysosomes in CD4+ T cells. A) CD4+ T cells were purified from healthy donors, activated, permeabilized and stained for CD63, cathepsin D, CTLA-4 and FasL. Primary antibodies were detected with fluorophore-conjugated anti-rabbit and isotype-specific anti-mouse secondary antibodies. Images are 3D reconstructions of serial z-sections taken by LSCM with the corresponding merged and DIC images are shown. Scale bar = 5μm. B) Quantification of colocalization (average r value) between cathepsin D and CD63 (n = 31), cathepsin D and CTLA-4 (n = 21), cathepsin D and FasL (n = 20), CD63 and CTLA-4 (n = 20), and CD63 and FasL (n = 20) is shown. Quantification was performed using single xy slices. Error bars are the SEM from four experiments performed using six individual donors. (TIF) [file ppat.1002226.s002.tif]

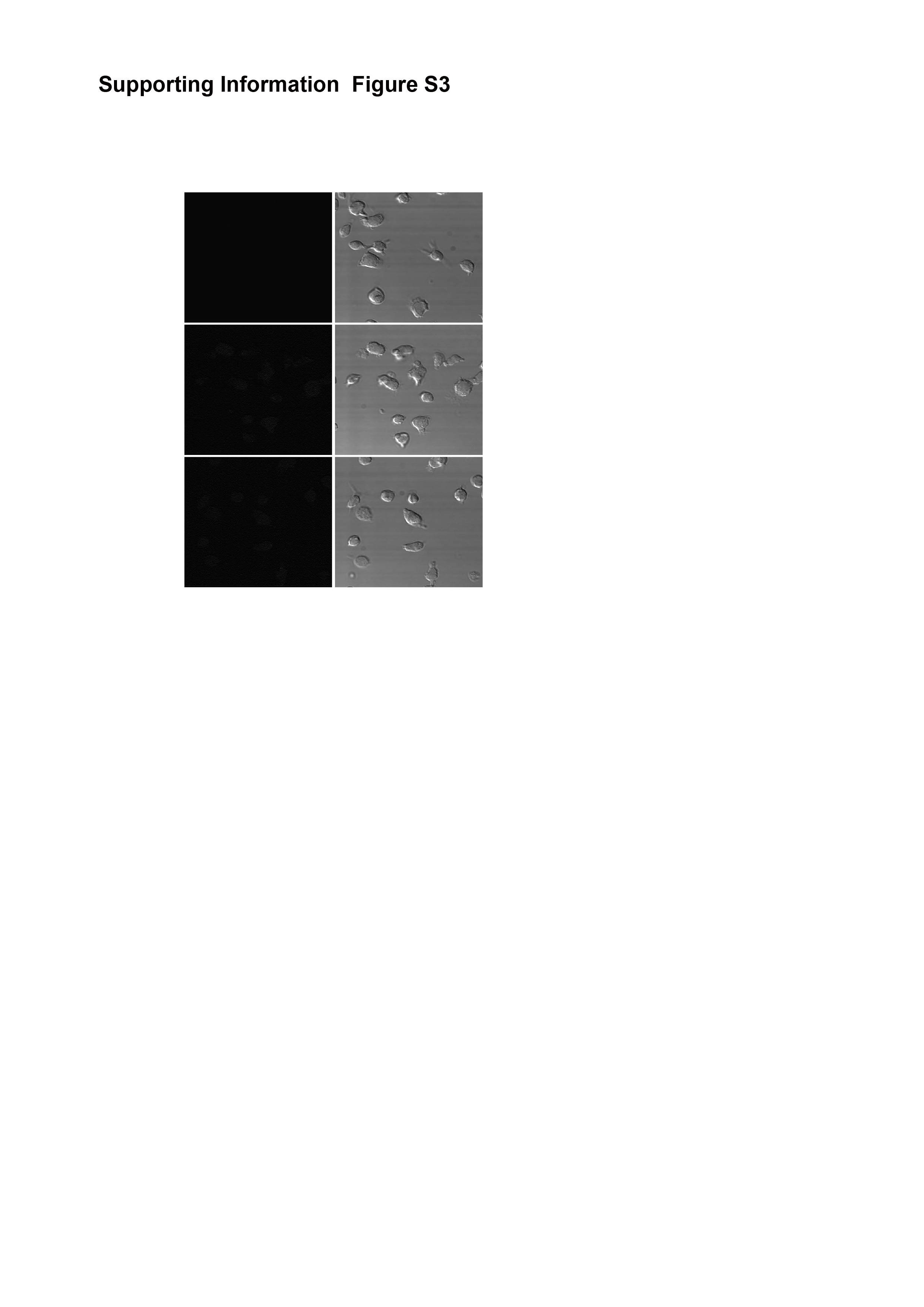

Supplement: Figure S3 — CD3 antibody is not internalized following T cell activation. CD4+ T cells were activated on plate-immobilized anti-CD3, detached with EDTA, washed, permeabilized and stained with isotype-specific anti-mouse IgG1 (top panel), IgG2a (middle) and IgG2b (lower) secondary antibody and analysed by LSCM. We did not detect intracellular CD3 staining, confirming the specificity of the CD63, CTLA-4 and FasL staining. (TIF) [file ppat.1002226.s003.tif]

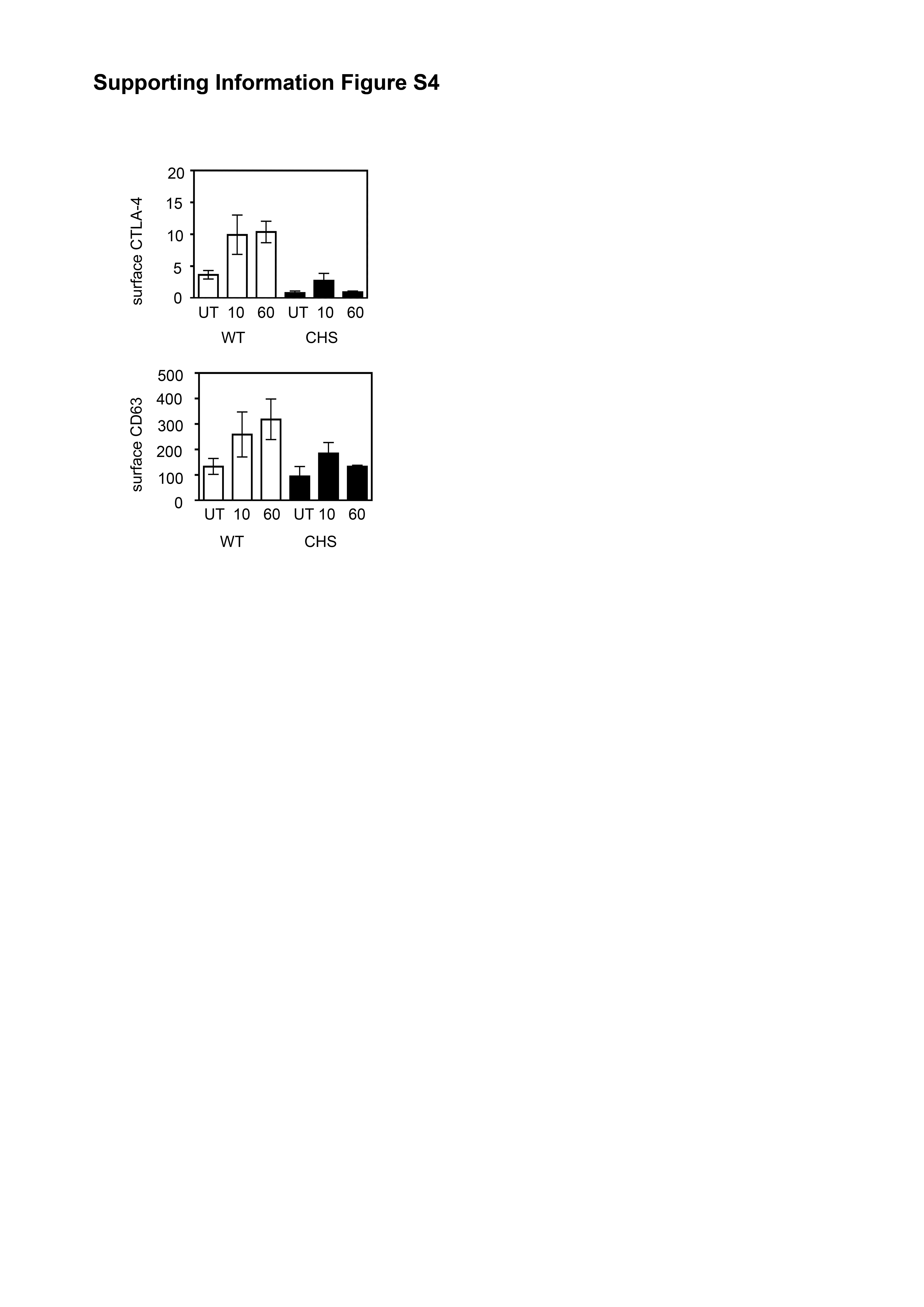

Supplement: Figure S4 — CHS CD4+ T cells show a defect in degranulation. Normal CD4+ T cells (white bars) or CHS CD4+ T cells (black bars) were either untreated (UT) or stimulated with PMA-ionomycin for 10 min or 60 min in the presence of mAbs specific for CTLA-4 or CD63. Cells were washed, stained with anti-mouse phycoerythrin-conjugated secondary antibody and surface expression of CTLA-4 (top panel) and CD63 (lower panel) was measured by flow cytometry. Graphs show the MFI and SEM of data combined from four experiments performed with four independent WT donors and two different CHS clones. (TIF) [file ppat.1002226.s004.tif]

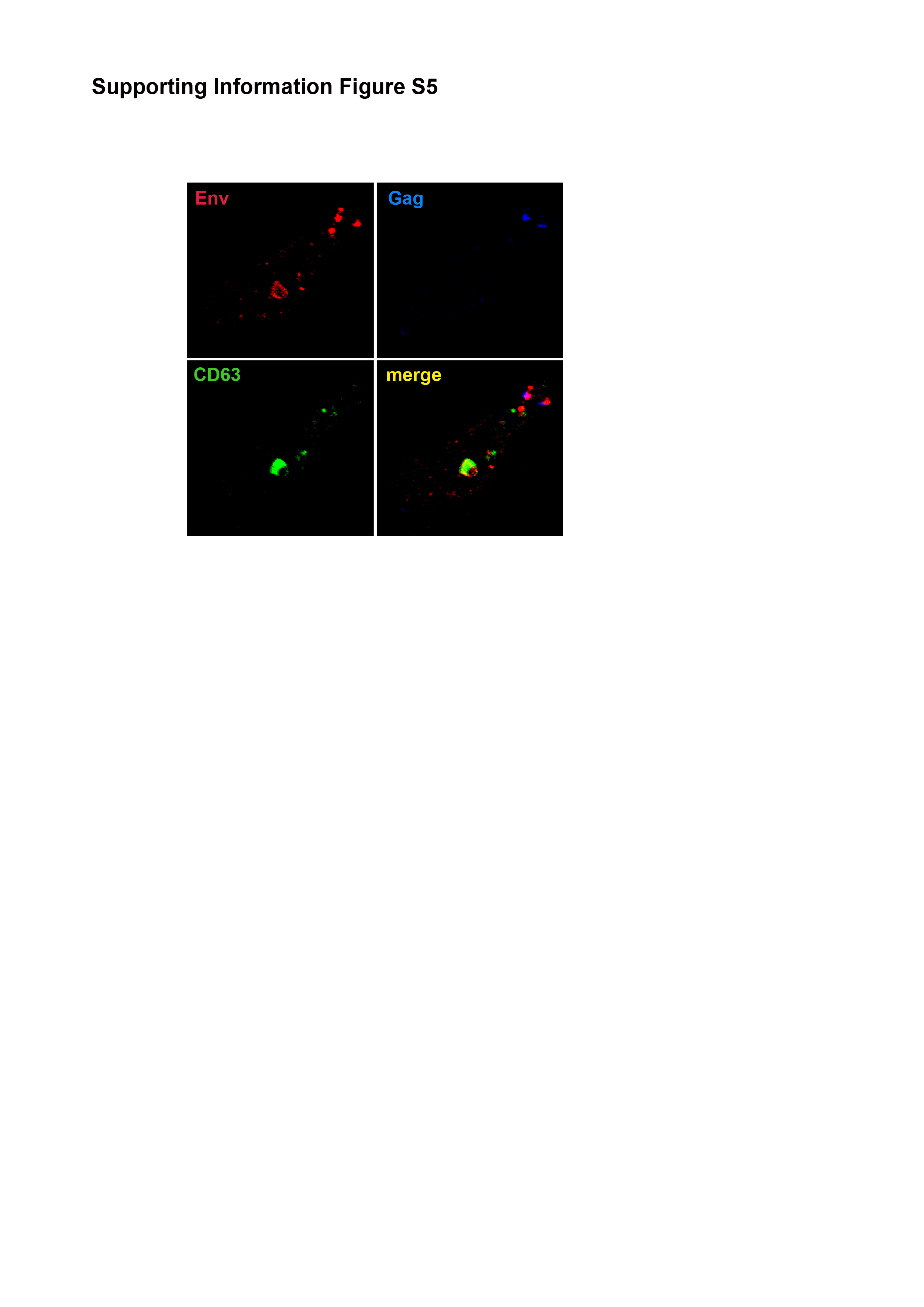

Supplement: Figure S5 — Gag does not localize to SL-related compartments in CHS CD4+ T cells. HIV-1-infected CHS cells were fixed, permeabilized and stained for Env (red), Gag (blue) and CD63 (green). Images are 3D reconstructed z-series and areas of colocalization are yellow. (TIF) [file ppat.1002226.s005.tif]

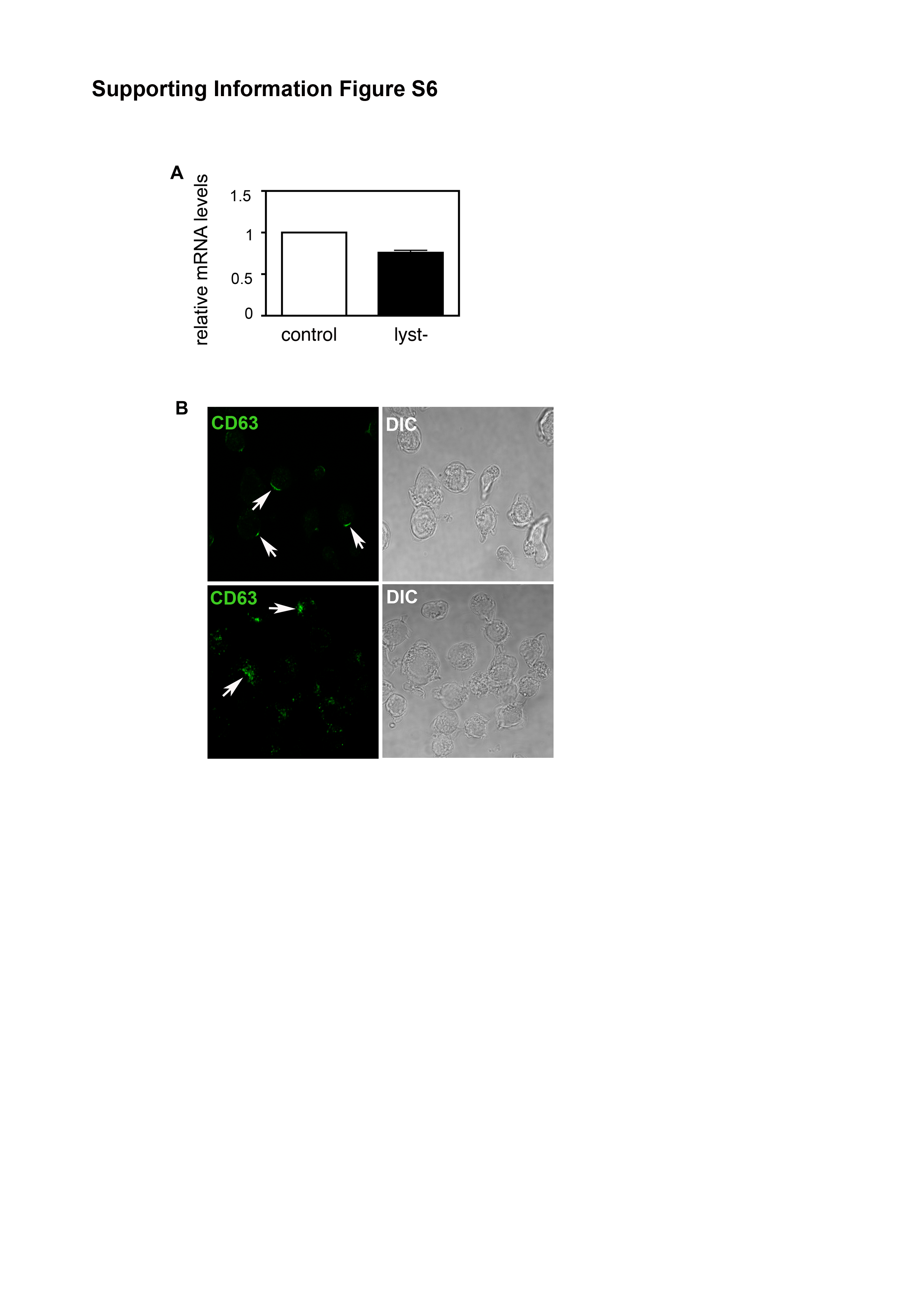

Supplement: Figure S6 — Validation of Lyst knockdown in Jurkat T cells. A) Total cellular RNA was extracted from Jurkat T cells stably expressing scrambled shRNA sequences (control cells, white bars) or Lyst-specific shRNA (black bars) and the relative quantification of Lyst mRNA determined by real-time reverse transcription PCR. Data are expressed as relative mRNA levels compared to control cells that are normalized to 1. Error bars represent the SEM from multiple experiments. B) Cells expressing scrambled shRNA (top panel) or Lyst-shRNA (bottom panel) were fixed, permeabilized and stained for CD63 (green). (TIF) [file ppat.1002226.s006.tif]

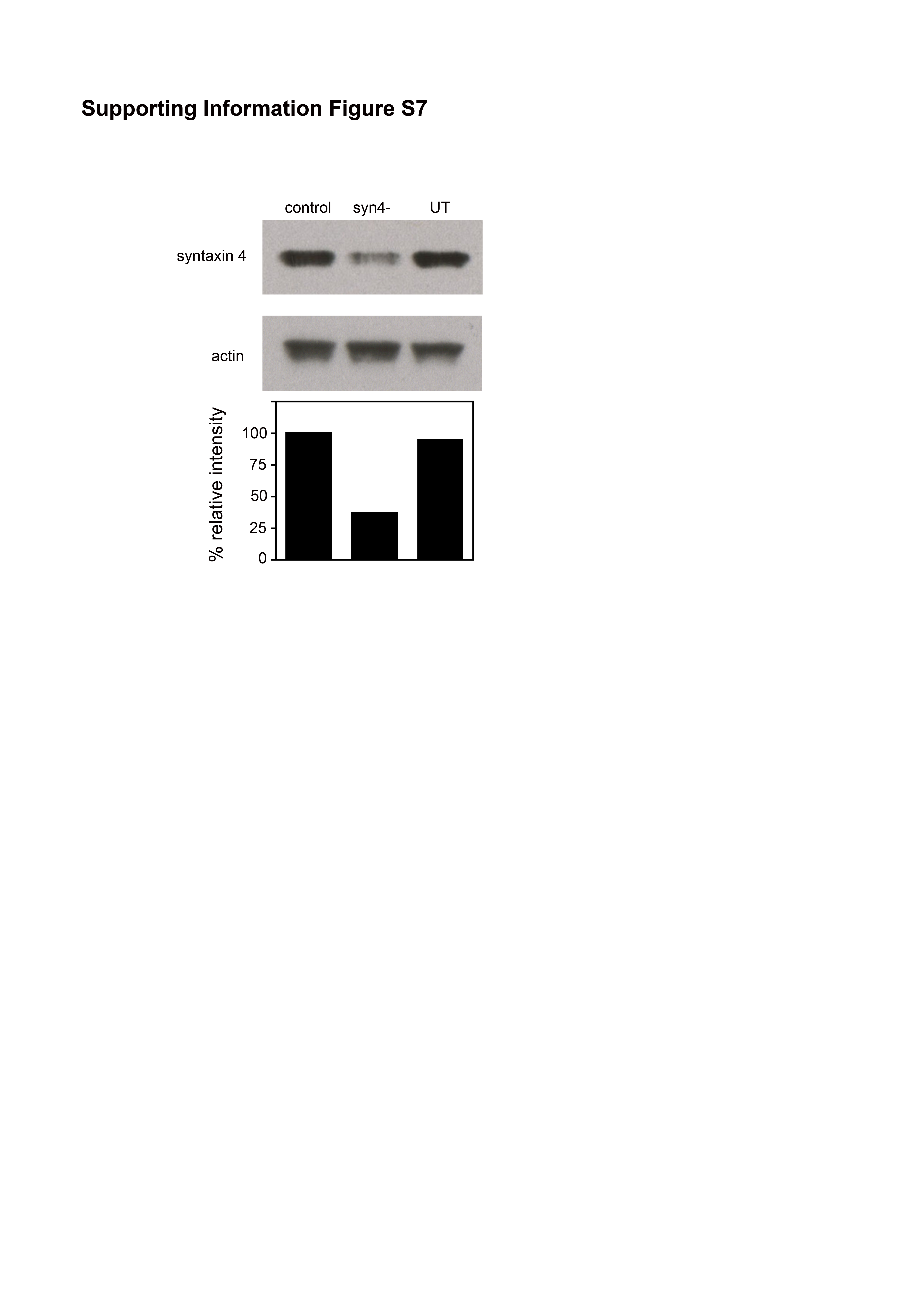

Supplement: Figure S7 — Quantification of syntaxin 4 knockdown in Jurkat T cells. Total cell lysates were separated by SDS-PAGE and Western blotting performed to determine the relative expression of syntaxin 4 protein in control-shRNA expressing cells, syntaxin 4-shRNA expressing cells and untreated Jurkat cells. The relative quantification of sytaxin 4 levels determined by densitometer analysis is shown. (TIF) [file ppat.1002226.s007.tif]
